# A Deterministic Analysis of Genome Integrity during Neoplastic Growth in *Drosophila*

**DOI:** 10.1371/journal.pone.0087090

**Published:** 2014-02-06

**Authors:** Cem Sievers, Federico Comoglio, Makiko Seimiya, Gunter Merdes, Renato Paro

**Affiliations:** 1 Department of Biosystems Science and Engineering, Swiss Federal Institute of Technology Zurich, Basel, Switzerland; 2 Faculty of Science, University of Basel, Basel, Switzerland; Ludwig-Maximilians-Universität München, Germany

## Abstract

The development of cancer has been associated with the gradual acquisition of genetic alterations leading to a progressive increase in malignancy. In various cancer types this process is enabled and accelerated by genome instability. While genome sequencing-based analysis of tumor genomes becomes increasingly a standard procedure in human cancer research, the potential necessity of genome instability for tumorigenesis in *Drosophila melanogaster* has, to our knowledge, never been determined at DNA sequence level. Therefore, we induced formation of tumors by depletion of the *Drosophila* tumor suppressor Polyhomeotic and subjected them to genome sequencing. To achieve a highly resolved delineation of the genome structure we developed the Deterministic Structural Variation Detection (DSVD) algorithm, which identifies structural variations (SVs) with high accuracy and at single base resolution. The employment of long overlapping paired-end reads enables DSVD to perform a deterministic, i.e. fragment size distribution independent, identification of a large size spectrum of SVs. Application of DSVD and other algorithms to our sequencing data reveals substantial genetic variation with respect to the reference genome reflecting temporal separation of the reference and laboratory strains. The majority of SVs, constituted by small insertions/deletions, is potentially caused by erroneous replication or transposition of mobile elements. Nevertheless, the tumor did not depict a loss of genome integrity compared to the control. Altogether, our results demonstrate that genome stability is not affected inevitably during sustained tumor growth in *Drosophila* implying that tumorigenesis, in this model organism, can occur irrespective of genome instability and the accumulation of specific genetic alterations.

## Introduction

Mechanisms maintaining genomic integrity are an essential part of the functional repertoire of any eukaryotic cell, as genome instability may not only have deleterious consequences for the affected cell but for the entire organism. The development of cancer, which is generally considered to be an acquired genetic disorder, has been associated with the gradual acquisition of genetic alterations. Genome instability, defined as an increased genomic mutation rate, is thought to enable this process, ultimately allowing cancer cells to acquire certain hallmark characteristics required for the multistep development of this disease [1]. However, a comprehensive characterization of the interdependence of genome instability and cancer development is still missing and may well depend on tumor and cell type. Whereas sequencing-based genome structure analysis revealed large mutability within certain human cancer genomes, suggestive for a general genome instability [2], [3], the data-based mathematical modeling and simulation of cancer initiation and progression suggests that other cancer types can develop at normal mutation rates [4], [5].

The fruit fly *Drosophila melanogaster* constitutes a genetically exceptionally well-defined tumor model, serving for the identification and characterization of numerous tumor suppressor genes and tumor relevant pathways [6]–[9]. In this model, the reduced genetic redundancy and biochemical diversity, compared to mammalian systems, facilitates the identification of cancer genes, since altering the activity of one gene is generally sufficient to initiate tumorigenesis. Hence, gene function and phenotype are directly correlated. However, whether tumor progression also requires the acquisition of additional genetic mutations has, to our knowledge, never been determined at the sequence level. Strikingly, a recent publication [10] demonstrates that induced chromosomal instability results in tumor formation and metastasis in a very short period of time in *Drosophila* epithelial cells, if apoptosis is blocked. This observation may indicate that a loss of genome integrity could indeed be a general feature of overgrowth in *Drosophila*, which remained unnoticed so far because some of the underlying changes might be subtle and therefore not detectable by methods of insufficient sensitivity. In addition, karyotype changes have been observed in tumorous tissue allografts from various mutants defective in genes that control asymmetric cell division [11], but whether these changes are directly involved in tumor progression has not been determined.

To assess whether neoplastic growth can occur irrespective of genome instability and the accumulation of specific genetic alterations in *Drosophila*, we set out to sequence and analyze the genome of a highly proliferative neoplastic epithelial tumor in order to identify structural variations (SVs); in this context defined as mutations that lead to changes in genome structure relative to a reference genome. To be able to determine SVs with high accuracy and at single base pair (bp) resolution we developed a deterministic SV detection algorithm, which benefits from the advancement of next-generation sequencing (NGS) technology and relies on long overlapping paired-end reads. The relevance of structural changes in cancer genomes has fostered the development of different strategies for the reliable detection of SVs [12], [13].

Split-read-based methods, for instance, aim to perform gapped alignments of sequences derived from SVs to reconstruct the altered genome structure enabling detection at single base pair (bp) resolution [14], [15]. However, as this approach was initially developed for longer Sanger sequencing reads, the application to NGS-derived reads is currently limited by the difficulty to align short reads unambiguously to the reference genome [13]. Another strategy involves paired-end reads generated by NGS. The overall procedure starts with the physical fragmentation of the genomic DNA. Importantly, the resulting DNA fragments are not of constant size and therefore follow a size distribution. A given number of bases are then sequenced from each end of the fragment resulting in a read pair. A discordant alignment is obtained if both reads of a pair align to the same strand or exhibit a mapping distance significantly different from the fragment size distribution. Such discordant read pairs are suggestive for the existence of a structural difference between the analyzed genome and the reference sequence, and are indicative for a broad range of SVs [16]–[19]. However, the sensitivity of this strategy is limited by the dependence on the fragment size distribution and identification of aberrant (discordant) read pairs requires a significant deviation from the expected fragment size. Therefore, the stochasticity of the fragment sizes translates into an uncertainty affecting SV detection, hampering detection of events within a certain size range. In addition, the paired-end strategy is generally not guaranteed to identify the breakpoints exactly.

A comprehensive analysis of genome structure, therefore, requires the combination of different strategies to compensate strategy-specific limitations [13], [20]. Here, we developed the Deterministic Structural Variation Detection (DSVD) algorithm which aims to combine advantages of split- and paired-end read-based approaches. This algorithm relies on long overlapping read pairs, a concept previously employed in sequence assembly [21]. By considering overlapping regions of the reads the DSVD-based analysis becomes entirely independent of the fragment size distribution and is able to determine SVs with high accuracy and at single bp resolution. In addition, DSVD provides a general graph-based framework used for the representation and detection of a broad class of SVs.

The Polycomb group (PcG) system, primarily involved in the maintenance of repressive chromatin states, contributes to the overall epigenetic regulation of genes. Its dysfunction has been associated with developmental disorder and various types of cancer in vertebrates [22]–[24]. Highly proliferative neoplastic epithelial tumor can be induced in *Drosophila* by loss of function of Polyhomeotic (Ph), one of the core components of the PcG system [25]. Here, we applied this tumor model in conjunction with the DSVD algorithm to assess whether neoplastic growth can occur irrespective of genome instability and the accumulation of specific genetic alterations in *Drosophila*.

## Results

### Overlapping paired-end genome sequencing of *polyhomeotic* tumors

**Figure 1:**
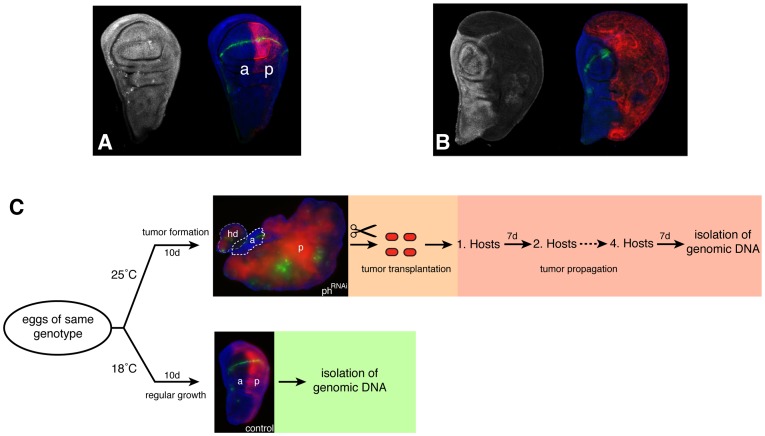
Depletion of *polyhomeotic (ph)* induces neoplastic tumors. A) Ph expression in the normal wing disc of third instar larvae (left) expressing the reporter *en-GAL4* 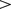 UAS-*myr-RFP*, UAS-*Dicer2*, *NRE:EGFP* (right). B) Downregulation of Ph induced by the RNAi reporter observed in the posterior compartment. Posterior compartment (red RFP) shows overproliferation phenotype (from [26]). C) Schematic outline of the experimental workflow. Eggs of the same genotype were developed at different temperatures. The tumor suppressor *ph* is specifically depleted (RNAi) at 25°C within the posterior compartment (p, RFP signal in red) of wing imaginal discs, leading to the formation of large tumors (upper). To allow for the accumulation of SVs, tumors are transplanted for a period of four weeks. At 18°C depletion of Ph is not sufficient to drive tumorigenesis (lower) and corresponding wing imaginal discs were used as control. Genomic DNA from both samples was isolated and subjected to paired-end sequencing. Notch-dependent EGFP expression (green) marks the boundary of the dorsal and ventral compartments. The white dashed outline marks the remnant anterior compartment (a) with normal Notch signaling along the dorsal/ventral boundary, while the grey dashed outline labels the haltere disc (hd).

To assess the relevance of genome integrity in *Drosophila* tumorigenesis we took advantage of a tumor model induced by downregulation of the tumor suppressor gene *polyhomeotic* in the posterior compartment of the wing disc [25], [26]. By using the GAL4-UAS system [27] a spatially and temporally controlled knockdown of tumor suppressor genes can be achieved. In our model GAL4 is specifically expressed within the posterior compartment of the wing imaginal disc (see Materials and Methods), leading to a posterior-confined expression of an *RFP* gene (UAS-*RFP*) and an interfering double stranded RNA targeting *ph* (UAS-*ph*RNAi). Figure 1A and B depict examples of both a wild type disc and a tumor, respectively. The penetrance of the phRNAi tumor phenotype, indicating the probability of tumor development, was approximately 0.15 (data not shown). *ph*RNAi tumors exhibit extensive morphological anomalies (Figure 1B and Figure S1A) and are characterized by fast growth, high cell density, and a loss of tissue polarity. Another transgene present in our tumor model is a Notch signaling pathway reporter expressing EGFP in response to Notch activity (*NRE:EGFP*). In wild-type discs, high Notch activity is restricted to the dorsal-ventral boundary (Figure 1A). However, upon Ph depletion we observed ectopic activation of the Notch signaling pathway (Figure 1B), which contributes to tumor growth [26]. The anterior compartment of the tumor, not affected by the RNAi, is clearly distinguishable from the tumorigenic tissue and Notch activity in this compartment resembles the normal pattern observed within the control (Figure 1C).

The acquisition of genomic aberrations during tumorigenesis is thought to be a gradual process, increasing in frequency as the cellular DNA damage response mechanisms become progressively affected by the onset of the disease. To increase the incidence probability of genomic aberrations in our tumor model, we prolonged the tumor growth period by repeated transplantation and culturing of allografts of the tumorigenic tissue in the abdomen of host flies (Figure S1B), over a total period of four weeks. A schematic representation of our experimental workflow is illustrated in Figure 1C. Notably, we observed a comparable growth characteristic and morphology in allografts of tumorigenic wing tissue from genetic *ph* knockout mutants (Figure S1C) ruling out tumor promoting capacities by secondary RNAi effects or by the stimulation of the RNAi machinery.

Next, we collected control material of the same genotype and from the same tissue but without neoplastic characteristics, to be able to distinguish somatic mutations and aberrations from germline events with high accuracy. For this purpose, we took advantage of the temperature sensitivity of the GAL4-UAS system. RNAi in our transgenic *Drosophila* model is temperature- and dosage-sensitive allowing for a broad range of allelic series (full knockdown at 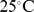 vs. partial knockdown at 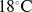) [26] to be generated. Accordingly, while depletion of Ph at 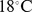 was insufficient to induce overproliferation, offspring larvae of the very same mothers kept at 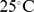 developed highly proliferative neoplastic tumors (Figure 1C). Thus, genomic DNA was isolated from control and tumor wing discs, developed at 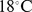 and 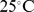, respectively. Next, we generated genomic DNA libraries exhibiting mean fragment sizes of approximately 250 bp (Figure S1D–E) and subjected these fragments to 150 bp overlapping paired-end sequencing, resulting in read pairs featuring a central overlap. Table 1 summarizes the sequencing results used for the subsequent analysis of the genome structure.

**Table 1:** Summary of the sequencing experiments and the seed-based alignment classification.

Sample	total number	concordant	discordant
control	113.1	23.3	2.3
tumor	90.9	27.6	3.2

Numbers refer to read pairs [million].

Since cancer progression has been associated with gross chromosomal rearrangements and aneuploidy, leading to copy number variations (CNV) of large genomic regions and karyotype changes [10], we used BICSeq, a CNV detection algorithm [28], to disclose such events in our samples. However, a global comparison of the tumor and the control coverage did not indicate the presence of large CNVs (Figure S2A). Another feature of cancer genomes, recently discovered in genome-wide studies of breast cancer, are mutational processes leaving specific signatures of base substitutions [3]. To investigate whether a similar process is activated during neoplastic growth in our tumor model, we analyzed the prevalence of single base substitutions [29] in the tumor versus control, but similarly, no substantial differences could be detected (Figure S2B). Therefore, we proceeded further and searched for tumor specific SVs at higher resolution using the DSVD algorithm outlined below.

### Overlapping read pairs allow exact alignment classification

**Figure 2:**
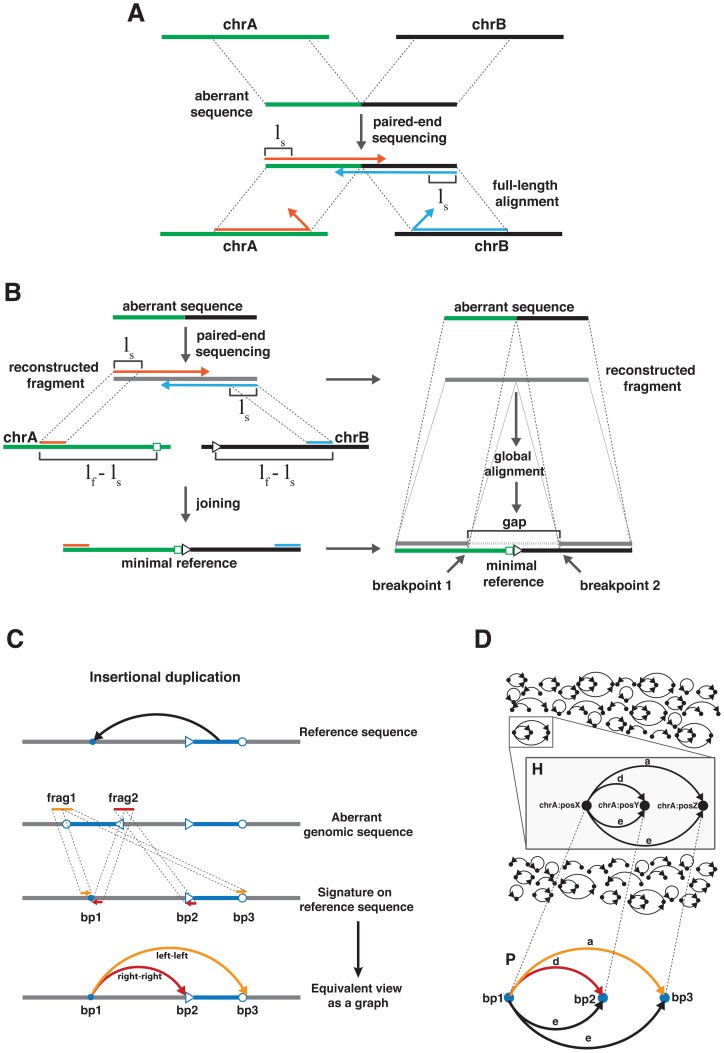
Schematic representation of the SV detection performed by DSVD. (A) Genomic rearrangements join different regions of the genome resulting in aberrant sequences. As a consequence, the full-length read alignment may fail. To avoid this problem, seeds of length 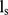, derived from the 5′-ends of the reads, are aligned instead. (B) Left: first, we constructed a minimal reference, i.e. the smallest possible region of the reference sequence possibly containing the reconstructed fragment. The construction requires the extension of the reference sequence in correct orientation, starting at the seed alignments, to a total length equal to 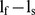 where 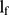 corresponds to length of the fragment sequence. The two extensions are ultimately joined to form the minimal reference. Right: next, for an exact identification of the breakpoints a global alignment of the reconstructed fragment sequence and the minimal reference is performed using the Needleman-Wunsch algorithm (see Materials and Methods). The gapped alignment is subsequently used to identify the breakpoint positions corresponding to the start and end position of the gap. (C) Schematic representation of an insertional duplication with inversion of the upstream inserted sequence (blue). Sequencing and processing of the two fragments (frag1 and frag2 in orange and red, respectively) spanning the boundaries of the aberrant region lead to the identification of the insertion site (bp 1) and the two virtual breakpoints (bp 2 and bp 3). The dashed lines connecting the reads of a pair derived from the fragments establish particular connections (Table 3) between different breakpoints resulting in an SV-type specific signature on the reference genome. In this example frag1 connects bp 1 and bp 3, approaching both breakpoints from the left. Similarly, frag2 establishes a connection between bp 1 and bp 2, approaching either genomic coordinate from the right. The connections formed by the read pairs can be represented explicitly by introducing directed edges between the different breakpoints. (D) Upper: schematic representation of the discordant graph representing all identified SVs. The example in the inset is similar to C. Lower: the prototype graph for the SV outlined in C. The graph structure represents the signature resulting from the duplication and inversion of the region between bp 2 and bp 3 followed by an upstream insertion at bp 1. Dashed lines highlight an isomorphism between the vertices of the prototype graph and the SV-representing subgraph, since the existence of an edge between two vertices in 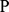 implies the existence of the same edge connecting the transformed vertices in 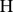.

The resolution of paired-end read-based strategies for SV detection is limited by the uncertainty within the fragment length, reflected by the fragment size distribution. However, advancements in NGS technology enable the generation of sufficiently long read pairs such that each read covers the central region of the fragment, while maintaining the fragment size distribution approximately constant. Accordingly, the centrally overlapping parts of read pairs can be used for the exact reconstruction of the fragment sequence [21]. Figure S3A shows a schematic representation of the procedure. As outlined below, the fragment sequence allows for both an exact evaluation of the mapping distance (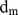) of the read pair alignment and a precise identification of the SV breakpoints. The detection and characterization of SVs requires the alignment of read pairs to a reference genome. As most informative fragments are derived from regions exhibiting systematic differences with respect to the reference genome, the presence of SVs is likely to impair the alignment and identification of discordant read pairs, ultimately leading to a loss of relevant information (see Figure 2A). To overcome this problem, we performed a seed-based alignment (Figure 2A) using seeds, subsequences derived from the high-confidence 5′-ends of the reads, similar to [30]. The choice of the seed length 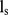 constitutes a tradeoff between ‘unique alignability’, defined as the probability that a randomly selected chromosomal subsequence of length 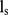 can be uniquely aligned to its origin, and recall, i.e. the ability to align the seed at all. Based on the evaluation of the unique alignability we determined a seed length 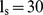 to be eligible for the *Drosophila* reference genome (Figure S3B–D). In order to account for SNPs and sequencing errors, seeds were aligned allowing one mismatch [31]. Furthermore, we required that both seeds of a read pair uniquely align to exactly one position in the genome. To identify discordant alignments the length 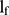 of the afore reconstructed fragment sequence is employed. Based on the mapping distance 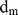 of the seed alignment the following classification rule is defined: if 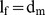 the alignment and hence the read pair is considered to be concordant, if 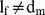 the alignment and hence the read pair is classified as discordant. In addition, the relative orientation of the seeds is taken into account during the classification. Consequently, by considering the fragment sequence and the seed alignments, discordant read pairs are identified at single base resolution. In addition to the single base-resolved classification of the read pairs, we employed the reconstructed fragment sequences for a precise characterization of SVs. Genomic rearrangements result in aberrant DNA sequences joining different parts of the genome (Figure 2A). The exact identification of breakpoints requires the mapping of the different fragment subsequences to their distinct origins within the reference genome. Based on the discordant seed alignments, we constructed a ‘minimal reference’ (Figure 2B, left), confining the search space to the smallest conceivable region of the reference genome possibly containing the fragment sequence. Subsequently, a pairwise global alignment involving the minimal reference and the reconstructed fragment sequence is performed resulting in a gapped alignment (see Materials and Methods). The genomic coordinates of the breakpoints can be inferred from start and end position of the gap (Figure 2B, right).

### Graph-based representation of structural variations

The applicability of different classes of graphs (e.g. undirected or de Brujin graphs) for a comprehensive representation and identification of SVs appears natural in this context and was successfully demonstrated before [32], [33]. In our approach, we integrate information represented by discordant read pairs, which relate different breakpoints, by using directed weighted multigraphs, thus, achieving a comprehensive representation of a broad range of SVs (see Materials and Methods). An exemplary SV is illustrated in Figure 2C.

To summarize and relate all breakpoints detected by all the discordant read pairs, we construct the so-called ‘discordant graph’ (Figure 2D, upper), which represents the entire information reflecting structural differences as detected by the sequencing experiment. In the discordant graph, vertices represent genomic coordinates of the breakpoints, edges correspond to connections established by read pairs (see enlargement in Figure 2D) and the weight of an edge is equal to the number of read pairs supporting this connection. The discordant graph is disconnected and consists of maximally connected subgraphs (connected components) corresponding to the different SVs (Figure 2D, upper).

**Figure 3:**
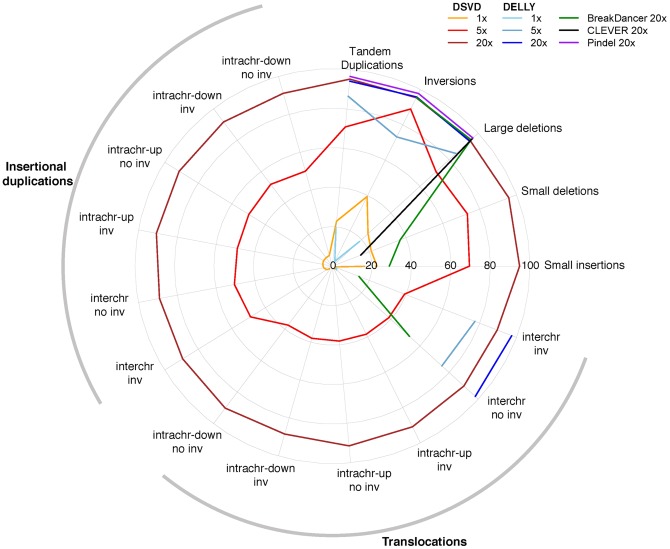
Performance comparison on simulated SVs. Summary of the recall [%] achieved by DSVD, DELLY, BreakDancer, CLEVER and Pindel on different SV types (Text S1). The coverages specified during the read simulations are indicated in the legend. intrachr  =  intra chromosomal; interchr  =  inter chromosomal; down  =  downstream; up  =  upstream; no inv  =  no inversion; inv  =  inversion.

The correct interpretation of these components is fundamental for the identification of the SV types they represent. The inset in Figure 2D illustrates a subgraph consisting of three breakpoints connected by edges of different types. This example shows that type and characteristics of the corresponding SV are difficult to infer by visual inspection. To overcome this problem, we defined so-called ‘prototype graphs’ and use them to search the discordant graph. For example, the prototype corresponding to an upstream insertional duplication with inversion (Figure 1C) is illustrated in Figure 2D (lower). The problem of identifying SVs can now be stated as follows: given a prototype graph, find all components within the discordant graph, which are isomorphic (‘equivalent’) to the prototype graph (see Materials and Methods). To illustrate this concept, an isomorphism between the graphs 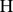 and 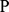 in Figure 2D is indicated. Since the prototype corresponds to a clearly defined SV, the mapping contains all information that are required to reconstruct the event exactly. In our example (Figure 2C–D) we can conclude that the genomic region between position Y and position Z was duplicated, inverted and upstream inserted at position X. Based on this framework, we defined a comprehensive set of prototype graphs (see Materials and Methods) and tested the performance of our algorithm on simulated data on 16 different SV types (see Text S1 and Table S4). Furthermore, we compared our algorithm to the recently published SV detection algorithms DELLY [33], BreakDancer [16], Pindel [14] and Clever [34]. The results of the simulation show that DSVD can identify all simulated SV types with high recall ranging from 0.88 (intrachromosomal translocation) to 0.96 (small deletions) for a coverage of 20. Although DSVD has a broader detection spectrum, the algorithm performs comparably to the other tested tools on the subset of SV types within their detection range (Figure 3). These simulation results indicate that the algorithm can be employed for the detection of SVs using sequencing data of the tumor and the control.

### Using concordant and discordant coverages to infer SV zygosity

The paired-end reads were subjected to the DSVD workflow outlined above. The maximum score values, obtained during the reconstruction of the fragment sequences, follow a bimodal distribution (Figure S4A). The mode around zero corresponds to non-overlapping read pairs derived from long fragments and the mode at one originates from overlapping read pairs. For subsequent analysis we only considered read pairs having a score equal to one, corresponding to a perfect overlap of at least 13 bases (see Materials and Methods). The reads pairs were subsequently aligned to the reference genome using a seed-based alignment (Figure 2A). The number of concordant and discordant alignments, obtained by the corresponding classification, is shown in Table 1. Then, we computed the overall read coverage – the genomic regions covered by concordant or discordant read pairs – to assess the fraction of the genome accessible to SV detection based on our sequencing data.

**Figure 4:**
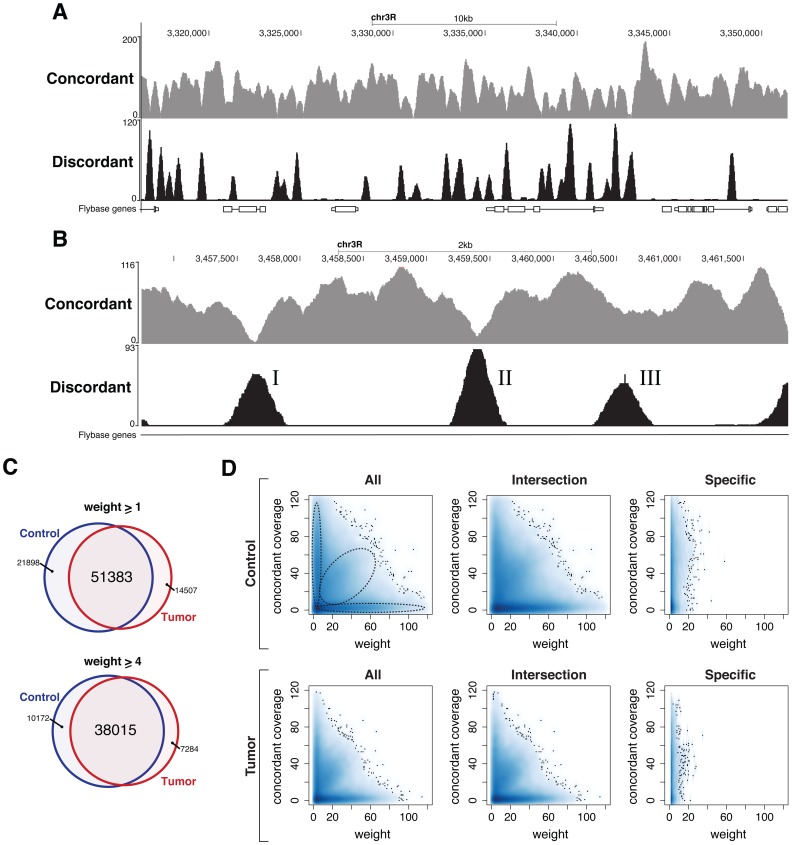
Concordant and discordant read coverage reveal extensive similarity between the tumor and control. (A) A genome browser view of representative 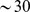 kb of the *Drosophila* reference genome. The tracks, denoted concordant and discordant, represent the total number of concordantly and discordantly aligning read pairs at a particular genomic position. (B) A browser view of homozygous and heterozygous SVs at higher resolution. In the case of homozygous SVs (I/II), the concordant coverage is decreasing to zero, as no wildtype allele is present anymore. In contrast, heterozygous events contain both a wildtype allele and an acquired SV, and are therefore characterized by a decrease within the concordant coverage to 50% (III). (C) Venn diagram representing the number of small insertions (of size 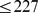 bp) identified within the tumor and the control with weight 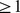 (upper) and weight 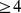 (lower), respectively. (D) Smoothed scatterplot representing the concordant coverage on the vertical axis and the discordant coverage (weight) on the horizontal axis for different subsets of small insertions. The three columns, from left to right, correspond to all small insertions (All), small insertions found in both genomes (Intersection) and small insertions specifically identified within the indicated genome (Specific), respectively.

The overall read coverage encompasses 91% to 96% of the different euchromatic regions (Figure S4B). A comparison between read coverage and unique alignability (93% to 96%) indicates that the largest proportion of the non-repetitive part of the genome is covered (Figure S4B). Due to their repetitive nature, heterochromatic regions showed a substantially lower overall coverage (21% to 53%, Figure S4C). In order to visualize the SVs called by DSVD in the genome browser, we computed the concordant and discordant coverages, from the corresponding alignments of the tumor and the control (Table 1). The concordant and discordant coverage exhibit opposite characteristics (Figure 4A), i.e. a decrease in the concordant coverage generally coincides with an increase in discordant coverage, which is indicative for underlying structural differences to the reference genome. Furthermore, consideration of concordant and discordant coverages allow to infer the zygosity of an SV: while homozygous SVs result in loss of both reference wildtype alleles and, hence, no concordant read-pairs are expected (Figure 4B, I and II), in heterozygous events one wildtype allele remains to contribute to the concordant coverage (Figure 4B, III).

### Genome instability is not a pre-requisite for neoplastic epithelial growth

Based on all discordant read pairs the discordant graph was constructed for the individual samples (Figure 2D). This led to identification of 311471 and 223285 distinct vertices (breakpoints) for the control and the tumor, respectively. To identify specific SVs within the discordant graphs we employed all previously defined prototype graphs in order to search the discordant graph for isomorphic subgraphs. Our search for small insertions, i.e. insertions which can be entirely characterized by a read pair (size 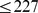), revealed 73281 and 65890 events in the control and the tumor, respectively, of which 51383 are detectable in both genomes allowing a tolerance of one base to account for equivalent alignments (Figure 4C, see Materials and Methods). Moreover, this overlap further increases when the minimum required weight (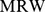, the number of read pairs supporting the same event) is raised from 1 to 4 (Figure 4C and Figure S5A). This tendency suggests that high confidence events are more likely to be present in both samples and, thus, to originate from germline rather than somatic mutations. The size distribution of insertions (Figure S5B) shows that most insertions are of length one, which is in good agreement with findings from rat, mouse and human, pointing to a median size of detected indels within introns of approximately 3 [35]. To assess the zygosity of small insertions, the number of concordant read pairs spanning the insertion site was considered (Figure 4D). This analysis identified three distinct classes (Figure 4D column 1–2) of small insertions characterized by: (i) high concordant and low discordant coverage (ii) comparable concordant and discordant coverage (iii) low concordant coverage and high discordant coverage. Insertions within the first class are likely to correspond to technical errors as they are characterized by low discordant coverage. In contrast, insertions belonging to the second and the third class are likely to correspond to heterozygous and homozygous events, respectively. Notably, the vast majority of small insertions specifically detected in either genome can be attributed to the first class (Figure 4D, last column) and therefore corresponds to low confidence events.

**Figure 5:**
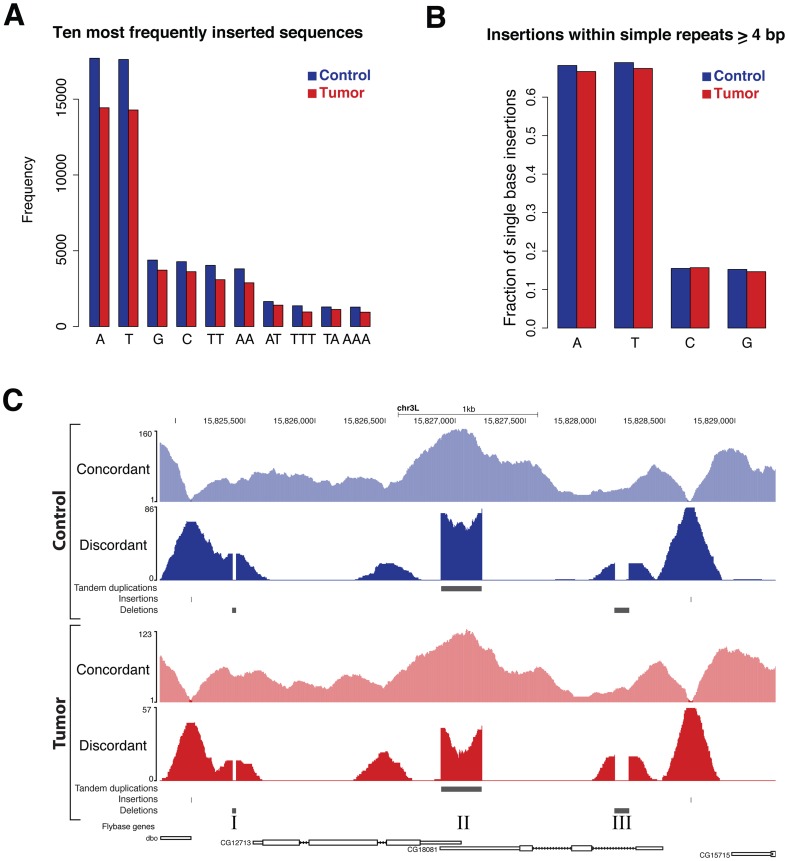
Genomic context analysis can indicate mutational mechanisms causing SVs. (A) DNA sequence and insertion frequency of the 10 most commonly inserted sequences identified within the control genome. For the tumor the tenth most frequently inserted sequence corresponds to CA with 897 insertions. For the sake of a clear representation the eleventh most frequently inserted sequence (AAA, 894 insertions) is shown. (B) The fraction of single base insertions within simple repeats consisting of the same base type, computed with respect to all single base insertions. Simple repeats of a minimum length of 4 were considered. (C) A genome browser view of a genomic locus containing two insertions (I/V), two deletions (II/IV) and one tandem duplication (III). As indicated by the discordant coverage and horizontal bars, these high-confidence SVs are both identified within the tumor and the control genomes, and have therefore been inherited from the parental strains.

Next, we sought to identify putative mechanisms responsible for the formation of insertions. To this extent, we analyzed inserted sequences and their context of insertion. We found that the 10 most prevalent insertions are similar in frequency and sequence identity within the tumor and the control (Figure 5A), and that most of these insertions are A/T rich. Subsequently, for each single base insertion, we computed the number of insertions within simple repeats of the same base (Figure S5C), and compared it to the total number of insertions. Our analysis revealed that the largest fraction of single base A or T insertions localizes within simple A or T repeats, respectively (Figure 5B). This indicates that context specific DNA replication errors, such as replication slippage [36], may be causative since this mechanism is known to cause indels within simple repeat sequences [37]. In addition, replication slippage is known to induce errors during PCR amplification [37]. It is therefore possible that aberrant DNA fragments are generated at low frequencies during PCR amplification of the genomic DNA library, possibly explaining the sample-specific low-confidence SVs (Figure 4D and Figure S5A).

Another mechanism resulting in permanent structural changes at the target site is the conservative transposition of DNA transposons. Following the excision of the transposon, a direct repeat remains at the target site [38]. To investigate the potential relevance of this mechanism, we identified all small insertions longer than two bp exhibiting perfect sequence identity to either flanking sequence, a requirement for a direct repeat (Figure S6A). The analysis revealed 15073 and 12060 such events in the control and the tumor, respectively, of which more than 50% was identified within low-complexity A/T-rich simple repeats (Figure S6B), complicating the distinction between replication slippage and transposition. However, by analyzing non-simple insertions (more than 2 different bases) we identified 5795 and 5375 insertions in the control and the tumor (4765 in common), respectively, which correspond to potential transposon-mediated events.

Using the deletion prototype graph, we identified 71969 deletions in the control and 64737 in the tumor, which showed similar characteristics to the insertions. In total 41696 deletions were detected within both samples (allowing one base tolerance) and high confidence events are more likely to be common to the two genomes (Figure S7A–B). The size distribution of deleted sequences indicates that single base deletions occur at highest frequency (Figure S5B). As compared to insertions, deleted sequences show a similar A/T enrichment and also occur preferentially within simple repeats (Figure S7C–D), suggesting a similar mechanism of formation. Examples of deletions found in the tumor and the control are shown in Figure 5C (I/III) and Figure S8A–B.

To assess the effect of small insertions and deletions on coding sequences of cancer-related genes, we analyzed indels (weight 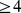) which were specifically identified in the tumor or the control using VariantAnnotation (Obenchain et al., VariantAnnotation: Annotation of Genetic Variants, package version 1.4.5). This analysis revealed approximately 2.5 times more genes potentially affected in the control than in the tumor (Table S1 and S2). In addition, GO term analysis of the affected genes did not result in any tumor specific term enrichments (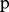-value 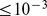) [39].

Searching for more complex SVs, revealed that tandem duplications and inversions occur at lower frequency and, similar to indels, the largest fraction of these events was common to both genomes (Figure S9). An exemplary tandem duplication is shown in Figure 5C (II). In addition, 15 and 13 translocations and insertional duplications were detected in the control and the tumor at a MRW of 2, respectively, of which 10 were common to both genomes.

**Figure 6:**
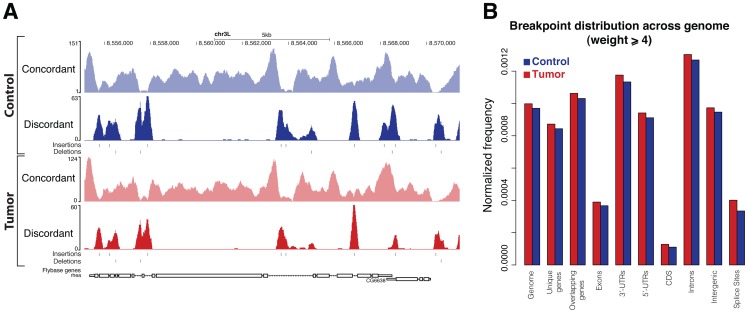
Coding sequences are less susceptible to SV accumulation. (A) Genome browser view depicting the concordant and discordant coverage of the control (blue) and the tumor (red) samples across two protein-coding genes, and identified SVs therein. The detected insertions and deletions localize outside of coding sequences, and affect introns, intergenic spaces and UTRs. (B) Genome-wide breakpoint distribution across distinct functional compartments. Different subsets of the genome were selected according to following characteristics: *genome* corresponds to the full-length genome; the *unique genes* do not share common positions with any other gene; *overlapping genes* are non-unique genes; *exonic* regions, containing *3′UTRs, 5′UTRs* and coding sequences (*CDS*) were obtained from the unique genes in order to avoid ambiguity; In addition, *intronic* and *intergenic* regions as well as donor/acceptor splice sites (*splice sites*) were considered. For each subset the number of contained breakpoints was computed and normalized to the total length.

Finally, visual inspection suggested that coding sequences are less susceptible to accumulate SVs, as they are characterized by a decrease in discordant coverage in comparison to non-coding regions (Figure 6A). To assess the breakpoint distribution globally, we partitioned the genome into distinct functional subsets and computed the normalized number of breakpoints therein. We found that exons, in particular the coding sequences, are generally less affected by SVs, whereas non-coding regions, such as intergenic or intronic sequences, exhibit a much higher predisposition for the accumulation of SVs (Figure 6B). These results are in accordance with previous studies [40]–[43] demonstrating the accumulation of SNPs and indels in intronic sequences of *Drosophila*.

### Experimental validation confirms SVs identified by DSVD

To experimentally validate our findings, we randomly selected 24 SVs of different type and size ranges, in addition to the two deletions (I/III) and the tandem duplication (II) in Figure 5C, for PCR-based analysis (Table 2 and Table S3). PCR was performed using genomic DNA of distinct individuals of the same genotype as the tumor tissue. Genomic DNA from the *Drosophila* reference genome strain was used as a control. In total, 23 reactions were confirmative as SVs were specifically detected within the tumor genome (Figure S10). However, the zygosity of four SVs differed from the predicted zygosity. Therefore, we extracted genomic DNA from ten individuals of the parental strains in order to retest these four events and, in addition, to retrace selected heterozygous SVs (Figure S11). Notably, and apart from one size deviation, all tested alleles were detected within parental individuals suggesting that the observed deviation from the predicted zygosity of SVs is most likely due to the non-isogenic background of the parental strains (Table 2) and Figure S11). This genetic heterogeneity can also explain that 4 out of 27 tested SVs were exclusively detected within the parental DNA, for which more individuals were tested.

**Table 2:** Summary of PCR-based validation experiments.

	n	Size range [bp]	Het	Hom	Confirmed
Small ins.	10	22–133	3	7	10
Deletions	12	22–245	8	4	12
Tan. dupl.	5	293–889	n.d.	n.d.	5

SVs are considered to be confirmed whenever at least one aberrant allele was detected either within *ph*-RNAi induced tumors or the parental strains irrespective of the zygosity. The size range of a tandem duplication corresponds to a single duplication event. n =  Number of tested events; Het  =  expected to be heterozygous; Hom  =  expected to be homozygous; Small ins.  =  Small insertions; Tan. dupl.  =  tandem duplications.

Furthermore, to validate the predicted breakpoint locations, we performed Sanger sequencing with selected homozygous SVs (Figure S10). Sequencing confirmed that DSVD was indeed able to identify SV breakpoints with single bp resolution (Table S5).

## Discussion

The model organism *Drosophila melanogaster* has contributed enormously to our understanding of the signaling pathways and cellular mechanisms required to control growth and development of multicellular organisms. Much of the mechanistic redundancy contributing to the proliferative homeostasis in mammals is lacking in *Drosophila*, which has promoted the development of tumor models applicable to study different aspects of human cancer. Such models have been equally useful to study onset and progression of hyper- and neoplastic growth, and metastasis, uncovering many of the critical contributions of the Wnt, Hippo, Notch, DPP, Hh and JAK-STAT pathways to tumorigenesis [9], [44]–[46]. However, while in human cancer research the sequencing of tumor genomes and the identification of SVs becomes increasingly a standard procedure, the relevance of genome instability during tumorigenesis in *Drosophila* is less well understood. Whereas the highly reproducible and rapid initiation of tumor suppressor knock-out/down mediated tumor growth may indicate that altered activity of a single cancer gene is sufficient to drive tumor progression in *Drosophila*, a systematic and highly resolved analysis of a *Drosophila* tumor genome has, to our knowledge, never been performed. In addition, effects on genome stability during prolonged tumor progression have not been studied. We therefore addressed the question whether sustained neoplastic growth can occur in *Drosophila* in the absence of genome instability and genetic mutation. On this account, we performed genome sequencing of tumors induced by the inactivation of a tumor suppressor (*polyhomeotic*) [26] and developed an algorithm for the analysis.

By employing long overlapping paired-end reads the Deterministic Structural Variation Detection (DSVD) algorithm allowed for a highly resolved genome structure analysis. The central overlap of the read pairs is employed to reconstruct the original fragment sequence which can be used to identify discordant alignments at single base resolution rendering the algorithm independent of the fragment size distribution. Additionally, the fragment sequence is used for the precise detection of breakpoints. The exact detection of breakpoints is complicated whenever multiple optimal and, therefore, indistinguishable alignments exist. In such cases DSVD exhaustively considers all potential breakpoints by constructing the corresponding graphs. A lower bound for the DSVD resolution is determined by the seed length 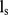. Hence, SVs separated by less than 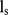 bases are likely to impede seed alignments and therefore to be missed. The general graph-based framework, provided by DSVD, allows for the representation and detection of a broad class of SVs irrespective of their sizes and can be easily extended to custom SV signatures. We tested the performance of DSVD on simulated data (see Text S1) for 16 SV types and compared it, on the commonly detectable SV types, to other recently published SV detection methods including DELLY. The results indicate that DSVD has higher recall at low coverage as the algorithm does not require read clusters to localize the SV and a single fragment spanning the breakpoint is generally sufficient for identification. Conversely, DELLY performs better on interchromosomal translocations. However, this higher recall can in part be explained, since DELLY does not attempt to distinguish between interchromosomal translocations and insertional duplications. DSVD achieves this classification by integrating additional evidences and by constructing more complex graphs. Overall, both algorithms are able to detect SVs with high recall and precision. As DSVD requires overlapping paired-end reads, the maximal fragment size is determined by technical capacities of the NGS technology. This has to be taken into account, when evaluating the required conditions for larger genomes, like the human genome. We furthermore expect the availability of longer reads, in conjunction with DSVD, to enable an improved SV detection within repetitive regions of the genome. Currently, DSVD is not designed to cope with complex SVs, e.g. nested deletions or insertions. Future improvements of the algorithm may involve the implementation and assessment of different alignment algorithms, such as AGE [15], to facilitate breakpoint detection in such cases.

In many human cancers the affected cells are characterized by the accumulation of genetic alterations leading to changes in gene structure, function or expression, which ultimately effect tumor suppressor and oncogenic pathways. In addition to complex patterns of SVs, often associated with increased genetic instability at late stages of cancer, cancer cells differ from surrounding healthy tissue by somatic mutations specifically affecting the CDS of genes involved in these pathways. In the tumor model described here in contrast, the largest fraction of all SVs was similarly detected within tumor and control. Hence, corresponding mutations are of germline and not somatic origin and were, therefore, not acquired during tumorigenesis. To assess if low frequency mutational processes may affect coding potential of tumor relevant genes possibly explaining tumor growth, we analyzed the effects of indels on coding sequences. However, not only was the total number of indels in coding sequences low, the vast majority of indels also exhibited a low allelic penetrance within the population and was predicted to cause non-synonymous mutations. In addition, we identified more than twice as many genes potentially affected within the control and the analysis of the affected genes revealed no specific GO term enrichment, rendering such mutational processes very unlikely to explain the highly reproducible overgrowth in our model. The larger number of potentially affected genes in the control sample can be explained by the higher number of individuals required to obtain enough genomic DNA for sequencing (20 wing discs from 10 offspring larvae), which increases the probability to detect low frequency alleles. The overall observed genetic variation within our laboratory stocks, with respect to the *Drosophila* reference strain, are explainable by temporal separation. Furthermore, the stocks have been kept constantly under laboratory conditions favoring the fixation of randomly acquired traits by genetic drift, as individuals are not exposed to external natural selection pressure anymore.

Altogether, our results demonstrate that, in *Drosophila*, sustained and rapid neoplastic overgrowth of epithelial tissue can indeed occur in the absence of somatic genome instability and genetic mutations. Thus, derailment of the cellular networks governing signaling pathways and growth control, either by direct protein-protein interactions or by epigenetic transcriptional control, is sufficient in this model organism to drive tumorigenesis, and permanent changes to the DNA sequence are not a prerequisite. This finding needs to be taken into consideration when interpreting mutant phenotypes in *Drosophila* cancer models. Nevertheless, genome structure analysis will be required in individual cases to exclude genome instability as significant contributor to overgrowth. To this end, this work provides both a sequencing strategy and an accompanying computational pipeline.

## Materials and Methods

### Induction of tumors and image recording

Tumors and mutant cell clones were induced, transplanted and documented essentially as described in [26]. The utilized fly strains are available from the Bloomington *Drosophila* Stock Center at Indiana University, USA, and the Vienna *Drosophila* RNAi Center, Austria. The parental *ph* RNAi stock with the VDRC transformant ID10679 and the genotype [*w1118; +; 10679/TM3*, *Sb*] was outcrossed to a strain of the genotype [*w1118; +; TM3*, *Sb/TM6*, *Tb*] to establish [*w1118; +; 10679/TM6*, *Tb*]. Males of this genotype were then mated to virgin females of the previously established fly strain [P{w+mC = UAS-Dcr-2.D}1, w1118; P{w+mW.hs = en2.4-GAL4}e16E, P{w+mC = UAS-myr-mRFP}1, P{w+m* = NRE-EGFP.S}5A; +] to obtain neoplastic tumor (at 25°C) or control tissue (at 18°C) samples for sequencing from the offspring larvae. To record images, wing imaginal discs were attached to the surface of glass cover slips. The images depict RFP in red, EGFP in green, and bright field images in blue. For RFP and EGFP maximum projections and for the bright field images average projections of horizontal optical sections are displayed with identical magnifications (additional details in [26]). Brightness and contrast were adjusted using Photoshop. To increase visibility of the discs shape in Figure 1, the discs outline in the blue channel (bright field images) were selected in Photoshop, the selections inverted and the backgrounds replaced with black.

### Preparation and sequencing of the genomic DNA library

Standard procedures were utilized throughout. Genomic DNA was isolated with the QIAamp DNA Micro Kit (Qiagen) and fragmented using the Bioruptor Plus (Diagenode); the Agilent High Sensitivity DNA Kit was used for quality control; libraries were prepared with the TruSeq DNA Sample Prep Kit (Illumina); to obtain a read length of 150 bases the TruSeq SBS Kit (v3) was used on a Illumina Rua HiSeq2000 equipped with a HiSeq Flow Cell (v3) and running the Illumina Pipeline Version 1.13.48.

### Reconstruction of the fragment sequence

In order to determine the position of the overlap between mates, the forward strand of read 1 and the reverse complement of read 2 were considered to compute a position-dependent overlap score 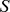 as:
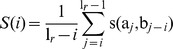
where



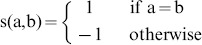
in which 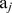 represents the base j (0-based offset) in read 1, 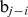 represents base 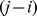 in the reverse complement of read 2, 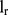 denotes the length the reads (here 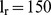) and 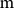 represents the minimum required overlap of both reads. The starting index of the overlap can be computed as:



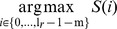



The probability of a false positive overlap is therefore equal to 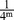. In this study a conservative value of 13 was chosen.

### Seed-based and global alignments

Seeds of the paired-end reads were aligned to *D. melanogaster* reference genome (dm3 BDGP Release 5) using Bowtie2 [47] with parameters -3 120 -N 1 -L 30 -k 11. Multiple alignments were discarded. The global alignment of the reconstructed fragment sequence was performed using the Needleman-Wunsch algorithm [48] with affine gap penalties [49] by setting gap opening penalty  = 10 and gap extension penalty  = 0.5.

### Representation of structural variants using graphs

A graph is defined as a pair 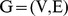. The elements of the vertex set 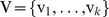 corresponds to distinct breakpoints. The edges in 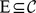 with 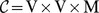 represent directed connections between the breakpoints established by a pair oriented from read 1 to read 2. 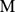 denotes the set of distinct edge types. An edge of type 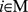 from node 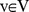 to 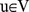 is denoted as 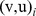 and the weight represents the number of read pairs supporting the same edge (not explicitly indicated for the sake of clarity in notation). The edge types belonging to 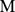 are shown in Table 3. Edge type 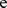 only occurs in combination with any other edge type. Since fragment orientation during sequencing is arbitrary, different edges may represent the same information. To account for such cases the following equivalence relations are defined on 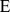:
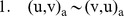


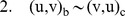


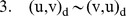



**Table 3:** Different directed edges belonging to 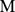. chr  =  chromosome.

element	orientation	domain of definition
a	left → left	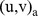 : 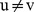
b	left → right	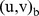
c	right → left	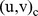
d	right → right	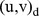 : 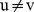
e	same chr. AND 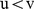	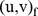 : 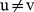

The existence of equivalent edges implies the existence of equivalent graphs. Hence, the equivalence class of a graph, i.e. the set of graphs corresponding to the same SV, was defined as follows. Consider the set of graphs 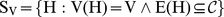, where 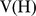 and 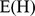 denote the vertex set and the edge set of 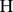, respectively. Two graphs 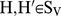 are equivalent if there exists a bijection 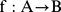 with 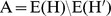 and 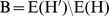, where 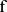 is defined by the equivalence relations (1–3) on the edges as:
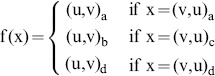



Multiple optimal alignments of the reconstructed fragment sequence and the minimal reference frequently occur within repeat sequences and hamper exact breakpoint identification. To illustrate this problem, consider the insertion of a single **A** into a homopolymer stretch of As such as AAA. Here, the following cases are indistinguishable: **A**AAA, A**A**AA, AA**A**A and AAA**A** and would result in the identification of different breakpoints. The problem of multiple optimal alignments becomes more relevant during the construction of higher order graphs. Indeed, identification of complex SVs require simultaneous identification of the same breakpoints from independent global alignments of different fragment sequences. Failure in doing so can lead to the identification of distinct breakpoints impeding the construction of the complete graph. To overcome this problem and ensure identification of the same breakpoint by different fragments, DSVD exhaustively constructs a graph corresponding to each optimal alignment (including a tolerance of 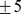 bases) resulting in collections of graphs representing equivalent SVs. From each collection one graph is selected. The selection is based on graph order and genomic position of the corresponding SV: higher order is prioritized over lower order and, in addition, graphs corresponding to central breakpoints within ranges of equivalent breakpoints are favored.

### Identification of structural variations using prototypes

Graph prototypes corresponding to well defined SVs were defined for: small insertions, deletions, tandem duplications, inversions, insertional duplications and translocations. For the last two SV types the following cases were exhaustively considered: interchromosomal vs. intrachromosomal, downstream vs. upstream and with and without inversion of the inserted sequence. The prototypes were then used to find all components of the discordant graph which are isomorphic to any graph within the equivalence class of the prototype. An isomorphism from a graph 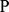 to a graph 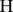 is a bijection defined on the vertex sets as 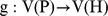 such that 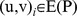 if and only if 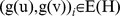. The mapping is bijective and structure preserving as edges of the same type connect corresponding vertices within the inverse image and the image.

### Implementation of the DSVD algorithm

The DSVD algorithm, detailed instructions as well as relevant documentation is available at:

http://www.bsse.ethz.ch/egg/software/index (11 June 2013, date last accessed). For all graph-related operations, DSVD depends on the python module NetworkX [50].

### Accession number

The sequence data of this study have been deposited at the short read archive (http://www.ncbi.nlm.nih.gov/sra) under accession no. SRP017639.

## Supporting Information

Figure S1 **Source material for paired-end sequencing.** (A) After dissection, wing imaginal discs from transgenic *Drosophila* larvae [*en*-GAL4, UAS-*myr*-RFP, *NRE:EGFP*] expressing an RNAi targeting *polyhomeotic* [*ph* ^RNAi^] at the indicated temperature, were attached to the surface of glass cover slips and bright field images recorded with a stereomicroscope. Wing imaginal discs, which developed at 18°C, are not of full wildtype morphology due to the weak impairment of Ph function. However, the tissues itself do not display any sign of overgrowth and are monolayer epithelia (transparent). At 25°C in contrast, largely overgrown tumors develop displaying characteristics of neoplasia (high cell density accompanied by a loss of tissue architecture and polarity). (B) Stereomicroscope images of a representative host fly carrying tumorous *ph*RNAi material; tu. The arrow marks the injection site, which is characterized by melanization. (C) Tumorigenic allografts, induced by the knockdown (*ph* ^RNAi^) or the knockout (*ph^Flp^*) of *ph*, have comparable growth characteristics and morphology, confirming the legitimacy of our RNAi-based tumor model. Cell clones (*ph^Flp^*) homozygous for mutations in both copies of ph (*ph-p* ^602^ and *ph-d* ^401^) [51] were induced somatically utilizing the FLP-FRT recombination system. (D–E) Size distributions of the genomic DNA libraries of the control (D) and the tumor (E) used for sequencing. The DNA fragments contain two sequencing adapters summing to 121 bp. The fragment size distribution is obtained by the according correction. Consequently, the modes of the fragment size distributions correspond to 272 bp (control, D) and 226 bp (tumor, E), respectively.
(TIF)

Figure S2
**CNV and SNP analysis.** (A) Size distribution of copy number variations (CNVs) called by the R package BICseq [28]. The function getBICseg was called using a window size of 200 bp and 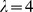. The results were filtered according to copy number ratio (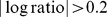) and p-value (p-value 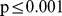). The CNVs were placed in genomic context using (Obenchain et al., VariantAnnotation: Annotation of Genetic Variants, package version 1.4.5). The following distribution was obtained: 78 introns; 13 splice site; 12 intergenic; 4 coding region; 0 within UTRs. No CNV longer than 8 kb was detected. (B) Venn diagram summarizing SNPs detected in the control and the tumor using BCFtools [29] and standard parameter settings.(TIF)

Figure S3
**Sequence reconstruction and evaluation of unique alignability.** (A) Schematic representation of the fragment sequence reconstruction. The index 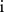 corresponds to the different alignments. For the quantification of sequence similarity an overlap score (see Methods in the main text) is defined. Depending on the degree of sequence similarity the score takes values of approximately zero in case random sequence similarity or values around one otherwise. The fragment is reconstructed by computing the overlap score for all alignments of a read pair. The alignment maximizing the score is returned and utilized to reconstruct the fragment sequence. (B) Schematic illustration of the set up used to assess the dependence of the unique alignability on the seed length 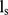. The reference genome was used to generate overlapping sequences of length 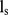 which were subsequently aligned back to the reference genome in order to evaluate uniqueness. For a fixed length 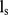 the sequences were chosen such that the entire genome was covered and two consecutive sequences are displaced by a single base. If 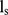 is chosen too short the seed may align to multiple positions in the reference genome and hence does not allow for an unambiguous identification of the origin of the read. However, larger values of 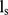 increase the probability that the seed contains the aberration and therefore fails to align at all. The resulting sequences were aligned to the reference genome by allowing one mismatch and used to determine the fraction of unique and multiple alignments. (C) and (D) show the fraction of unique and multiple alignments within the euchromatic and heterochromatic parts of the genome, respectively, as a function of 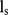 for 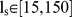. (C) Overlapping sequences of length 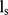 were generated (as illustrated in B) and aligned to the reference genome. The fraction of unique (blue) and multiple (orange) alignments, obtained for the euchromatic chromosomes 2L, 2R, 3L, 3R, 4 and X, are summarized as distributions and plotted as a function of 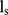. The solid and dashed lines represent the maximal and minimal fraction of unique alignments, respectively, resulting from 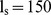 on the different chromosome. The increase of 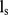 leads to an increase of the fraction of uniquely alignable sequences accompanied by a drop in the fraction of multiple alignments. Within the considered size interval the fraction of uniquely alignable sequences exhibits a rapid convergence close to the maximal achievable value in this setting obtained by considering 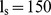, which corresponds to the full-length read. The consideration of 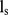 values larger than 30 does not substantially improve the unique alignability within the euchromatic regions. (D) Same as C, except results of the heterochromatic chromosomal regions including 2LHet, 2RHet, 3LHet, 3RHet, XHet and YHet are shown. The heterochromatic parts of the genome exhibit substantially less unique sequence characteristics. Considering sequences of 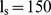 results in a maximal unique alignability close to 70%.(TIF)

Figure S4
**Assessing the characteristic uniqueness of the reference genome.** (A) Bimodal distribution of the maximum score values obtained from reads pairs of the tumor and the control. The mode around zero corresponds to read pairs without sequence similarity derived from fragments longer than 287 bp as a minimum overlap of 13 was required (Methods). The mode around one corresponds to overlapping read pairs. Mismatches within overlapping regions can lead to score values close to and smaller than one. The probability mass distribution between the two modes is different between the tumor and the control: the first mode is less pronounced within the tumor, where more probability mass localizes at one. This can be explained by the fact that the tumor derived fragments are on average shorter than fragments from the control sample, resulting in a larger fraction of overlapping read pairs ultimately contributing to the second mode (Fig. S2D–E). (B, C) Assessment of the read coverage within euchromatic (B) or heterochromatic (C) regions obtained from each sequencing experiment. Bars indicate the total length of the chromosomes, the unique alignability resulting from 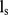 = 30, and the length of the genomic region covered by concordant or discordant reads, both in the control and the tumor.(TIF)

Figure S5
**Characterization of the small insertions.** (A) The top panel represents the total number of identified small insertions as a function of the minimum required weight (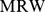), i.e. the number of read pairs supporting the same event. The lower panel shows the fraction of small insertions present in both samples as a function of the 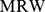. (B) Size distribution of recognized insertions/deletions. Positive and negative integers correspond to small insertions and deletions, respectively, of corresponding size. (C) Exemplary browser view of a T insertion within a simple T repeat of length 5. The same insertion was detected within the tumor and the control.(TIF)

Figure S6 **Small insertions can result from the conservative transposition.** (A) Exemplary browser view of an insertion of length 5 resulting in a non-simple directed repeat. The same insertion was detected within the tumor and the control. (B) The 10 most frequent insertions of minimum length 3 resulting in the formation of a directed repeat in the control and the tumor.
(TIF)

Figure S7
**Characterization of the deletions.** (A) Venn diagram representing the number of deletions identified within the tumor and the control. Upper panel corresponds to deletions of weight 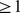, lower panel shows deletions of weight 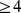. (B) The top panel represents the total number of identified deletions as a function of the minimum required weight (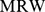). The lower panel shows the fraction of deletions present in both samples as a function of the 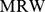. (C) Frequency and DNA sequence of the 10 most commonly deleted sequences in the tumor and the control genomes. (D) The fraction of single base deletions within simple repeats consisting of the same base. Simple repeats of minimum length of 4 bp were considered for the analysis. The fraction is computed with respect to all corresponding single base deletions.(TIF)

Figure S8 **Examples of homozygous and heterozygous deletions.** (A) Genome browser view of a homozygous deletion identified within both samples. In both cases the concordant coverage decreases towards zero. (B) Genome browser view of a heterozygous deletion identified within both samples. In both cases the concordant coverage shows a decreases across the affected region.
(TIF)

Figure S9
**Characterization of inversions and tandem duplications.** The top panel represents the total number of identified inversions (A) and tandem duplications (B) as a function of the 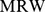. The lower panel shows the fraction of the according SV present in both samples as a function of the 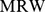. In both cases 10 base tolerance per breakpoint was considered, as the formation mechanism of such events may involve repetitive sequences [38], leading to ambiguity in the breakpoint detection caused by multiple optimal alignments. The large variance in the lower panel of (A) is caused by the low absolute number of inversions, e.g. for a weight 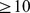 we observe 10 and 5 inversions for control and tumor, respectively.(TIF)

Figure S10
**PCR-based validation of small insertions, deletions and tandem duplications.** (A) PCR primers were designed to test different homozygous and heterozygous SVs. The selected SVs and their characteristics are described in Table S3. The numbering of the lanes corresponds to the SV IDs (Table S3). PCR was performed with genomic DNA of a Tumor (t) (obtained from the same *ph* RNAi strain as the tumors used for sequencing) and the reference strain (r) used to generate the *D. melanogaster* reference genome. In case of heterozygous events two PCR products are expected. (B) Same as (A), where the SVs indicated in Figure 4C have been tested. 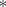 indicates SVs further confirmed using Sanger sequencing (Table S5); 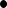 indicates heterozygous SVs that were also analyzed in the parental strains.(TIF)

Figure S11 **PCR-based validation of selected SVs in the parental strains.** SVs that failed to be detected in Figure S10 (lanes 13, 18, 22 and 27) in addition to selected heterozygous events (Figure S10) were further tested in the parental strains. PCR was performed with genomic DNA of five individuals of each parental strain, indicated as en (engrailed) and ph (polyhomeotic, see Methods for details) and with genomic DNA from the reference strain (r). The observed size of the tandem duplication (27, corresponding to event II in Figure 4C in the main text) is approximately three times the size of a single duplication event. Since single and multiple tandem duplications cannot be distinguished based on the signature on the reference genome, this size increase possibly indicates three consecutive duplication events.
(TIF)

Table S1
**Effects on coding sequences caused by small insertions of weight (w) 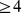 specifically found within the tumor or the control.** Indicated are genomic coordinates of the insertion (chr, pos), gene strand (strand), discordant coverage (w) as well as concordant coverage at the breakpoint (conc. coverage), location within the coding sequence (CDS location), Flybase gene ID (Gene ID) and the consequence of the insertion on the amino acid sequence (Consequence).(PDF)

Table S2
**Effects on coding sequences caused by deletions of weight (w) 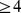 specifically found within the tumor or the control.** Indicated are genomic coordinates of the deletion (chr, start, end), gene strand (strand), discordant coverage (w) as well as concordant coverage at the breakpoints (conc. cov. 1, conc. cov. 2), location within the coding sequence (CDS start, CDS end), Flybase gene ID (Gene ID) and the consequence of the deletion on the amino acid sequence (Consequence).(PDF)

Table S3 **PCR-based validation of small insertions (ID 1–10), deletions (ID 11–20; 25–26) and tandem duplications (ID 21–24; 27).** Homozygous (Hom) and heterozygous (Het) events were selected. The ‘length’ corresponds to the size of the event. The ‘size on ref.’ shows the expected product size of PCR product as estimated from the reference genome. The ‘corrected size’ represents the expected product size in a sample containing the SV (for tandem duplications, this corresponds to the size expected from a single duplication event). small ins  =  small insertion; del  =  deletion; tan dup  =  tandem duplication; SV  =  structural variant; FWD  =  forward; REV  =  reverse.
(PDF)

Table S4
**Simulation results.** 1000 structural variations of the specified type were generated. 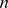 refers to the total number of recalled events, whereas 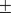 refers to the tolerance used to evaluate SV calls with respect to the genomic coordinates of the generated SV. For translocations marked with 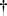, the number of insertional duplications are indicated in parenthesis as they correspond to events matching a partial signature. BD  =  BreakDancer.(PDF)

Table S5 **DNA sequences of selected SVs obtained through Sanger sequencing of the PCR products indicated Figure S8.** SV sequences (reported in the second column) are indicated in blue. Deletions are additionally crossed by a line. Flanking sequences are shown in black. Bases in red correspond to the 5 positions adjacent to the SV. Sequencing results (third column) show the Sanger sequencing results. Red bases across columns indicate corresponding positions.
(PDF)

Text S1 **Simulation of SVs for comparison of DSVD with other SV detection algorithms.**
(PDF)
