# Supplementary figures and images for: A Deterministic Analysis of Genome Integrity during Neoplastic Growth in Drosophila
Source: PLoS One. 2014 Feb 6;9(2):e87090. doi: 10.1371/journal.pone.0087090 (PMC3916295; doi:10.1371/journal.pone.0087090)

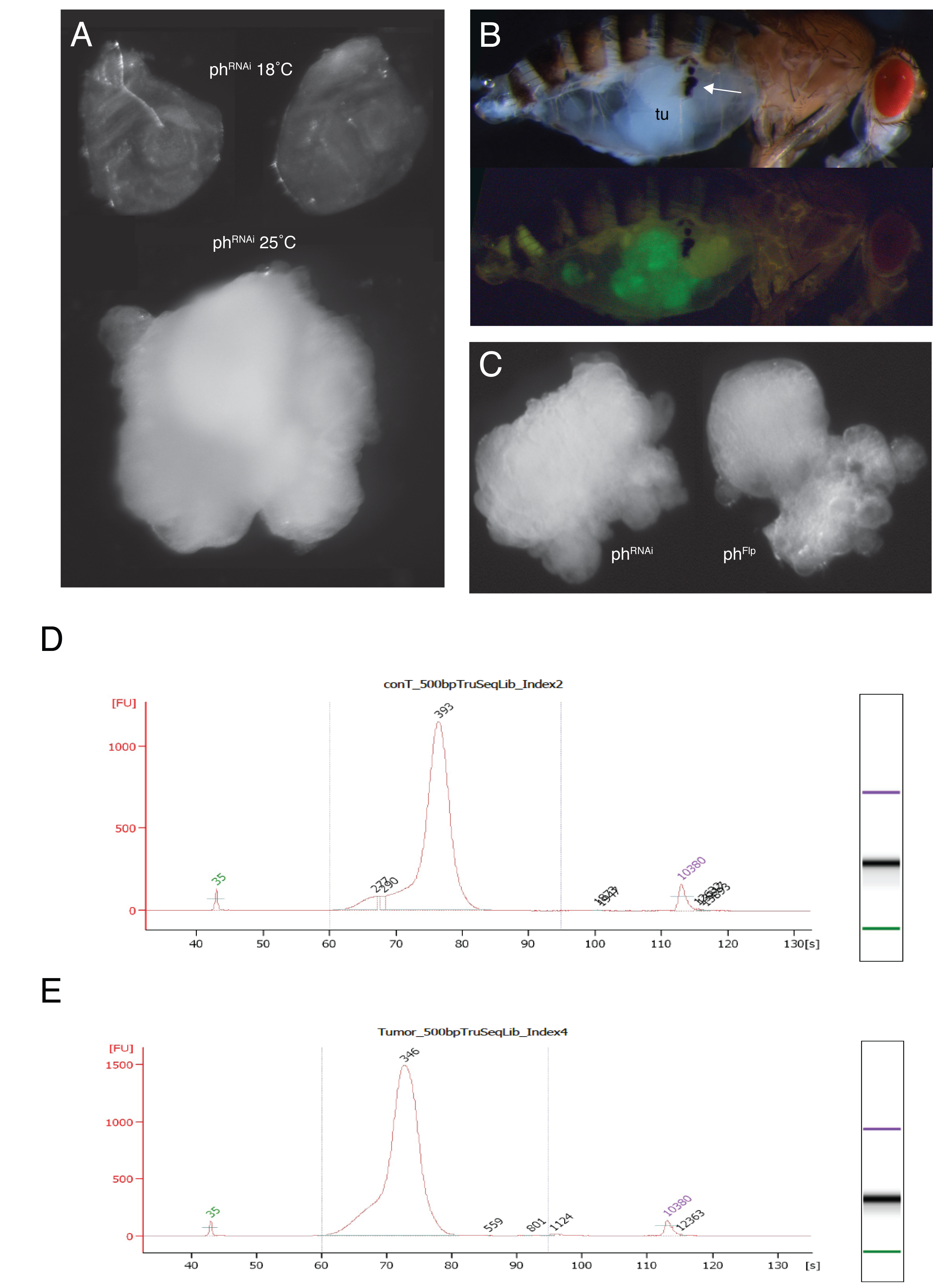

Supplement: Figure S1 — Source material for paired-end sequencing. (A) After dissection, wing imaginal discs from transgenic Drosophila larvae [en-GAL4, UAS-myr-RFP, NRE:EGFP] expressing an RNAi targeting polyhomeotic [ph RNAi] at the indicated temperature, were attached to the surface of glass cover slips and bright field images recorded with a stereomicroscope. Wing imaginal discs, which developed at 18°C, are not of full wildtype morphology due to the weak impairment of Ph function. However, the tissues itself do not display any sign of overgrowth and are monolayer epithelia (transparent). At 25°C in contrast, largely overgrown tumors develop displaying characteristics of neoplasia (high cell density accompanied by a loss of tissue architecture and polarity). (B) Stereomicroscope images of a representative host fly carrying tumorous phRNAi material; tu. The arrow marks the injection site, which is characterized by melanization. (C) Tumorigenic allografts, induced by the knockdown (ph RNAi) or the knockout (phFlp) of ph, have comparable growth characteristics and morphology, confirming the legitimacy of our RNAi-based tumor model. Cell clones (phFlp) homozygous for mutations in both copies of ph (ph-p 602 and ph-d 401) [51] were induced somatically utilizing the FLP-FRT recombination system. (D–E) Size distributions of the genomic DNA libraries of the control (D) and the tumor (E) used for sequencing. The DNA fragments contain two sequencing adapters summing to 121 bp. The fragment size distribution is obtained by the according correction. Consequently, the modes of the fragment size distributions correspond to 272 bp (control, D) and 226 bp (tumor, E), respectively. (TIF) [file pone.0087090.s001.tif]

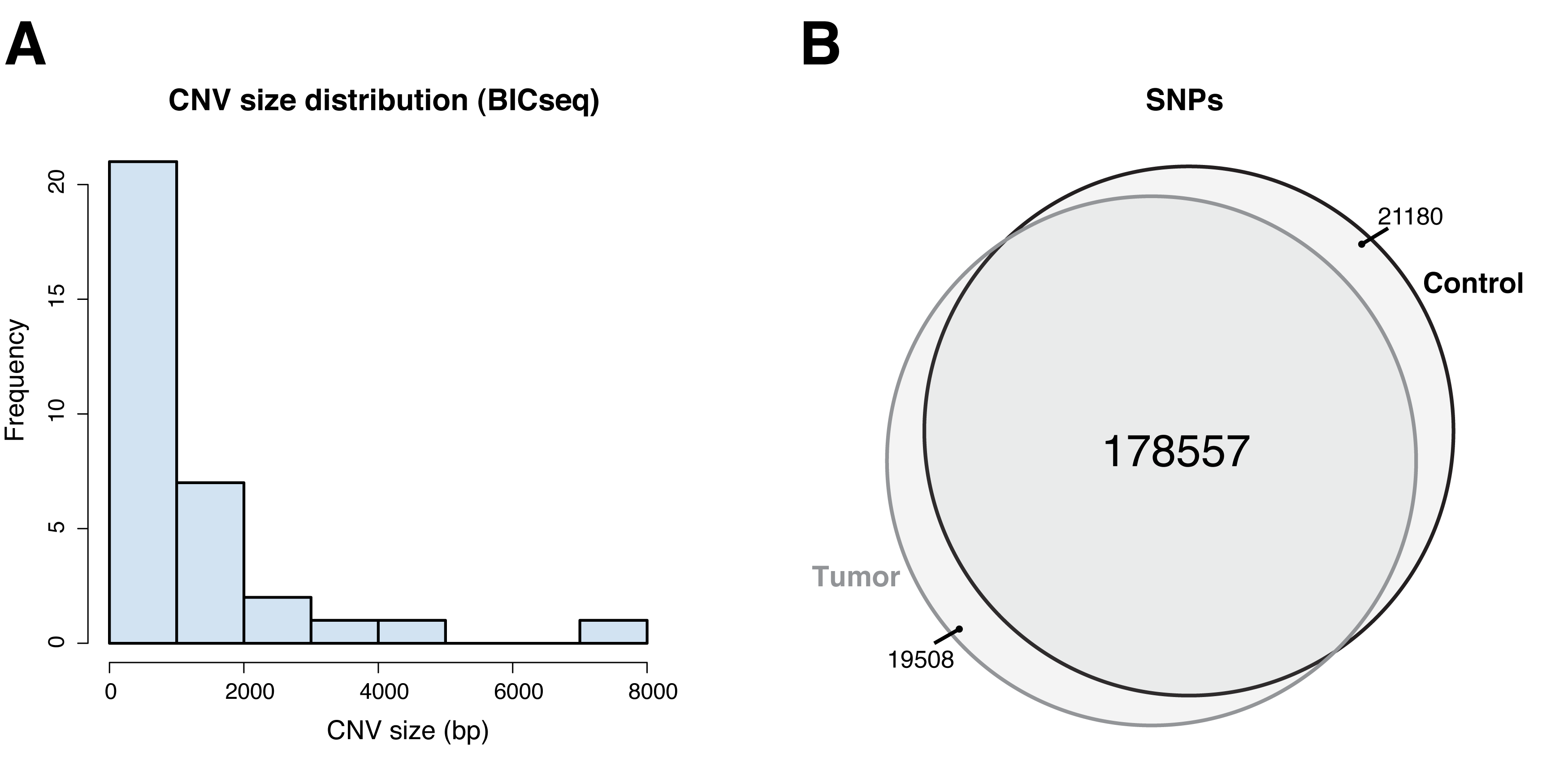

Supplement: Figure S2 — CNV and SNP analysis. (A) Size distribution of copy number variations (CNVs) called by the R package BICseq [28]. The function getBICseg was called using a window size of 200 bp and . The results were filtered according to copy number ratio () and p-value (p-value ). The CNVs were placed in genomic context using (Obenchain et al., VariantAnnotation: Annotation of Genetic Variants, package version 1.4.5). The following distribution was obtained: 78 introns; 13 splice site; 12 intergenic; 4 coding region; 0 within UTRs. No CNV longer than 8 kb was detected. (B) Venn diagram summarizing SNPs detected in the control and the tumor using BCFtools [29] and standard parameter settings. (TIF) [file pone.0087090.s002.tif]

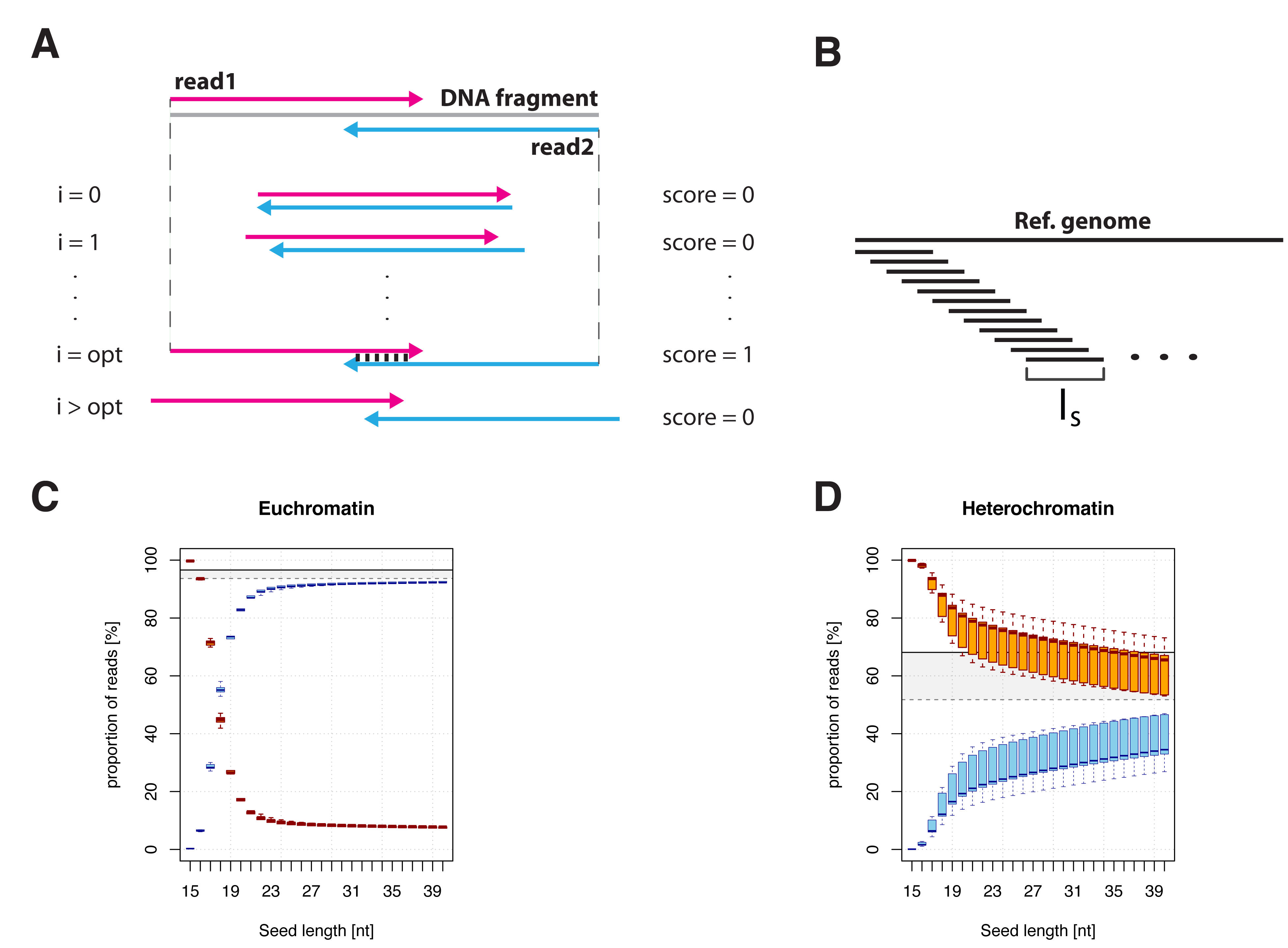

Supplement: Figure S3 — Sequence reconstruction and evaluation of unique alignability. (A) Schematic representation of the fragment sequence reconstruction. The index corresponds to the different alignments. For the quantification of sequence similarity an overlap score (see Methods in the main text) is defined. Depending on the degree of sequence similarity the score takes values of approximately zero in case random sequence similarity or values around one otherwise. The fragment is reconstructed by computing the overlap score for all alignments of a read pair. The alignment maximizing the score is returned and utilized to reconstruct the fragment sequence. (B) Schematic illustration of the set up used to assess the dependence of the unique alignability on the seed length . The reference genome was used to generate overlapping sequences of length which were subsequently aligned back to the reference genome in order to evaluate uniqueness. For a fixed length the sequences were chosen such that the entire genome was covered and two consecutive sequences are displaced by a single base. If is chosen too short the seed may align to multiple positions in the reference genome and hence does not allow for an unambiguous identification of the origin of the read. However, larger values of increase the probability that the seed contains the aberration and therefore fails to align at all. The resulting sequences were aligned to the reference genome by allowing one mismatch and used to determine the fraction of unique and multiple alignments. (C) and (D) show the fraction of unique and multiple alignments within the euchromatic and heterochromatic parts of the genome, respectively, as a function of for . (C) Overlapping sequences of length were generated (as illustrated in B) and aligned to the reference genome. The fraction of unique (blue) and multiple (orange) alignments, obtained for the euchromatic chromosomes 2L, 2R, 3L, 3R, 4 and X, are summarized as distributions and plotted as a function of . [file pone.0087090.s003.tif]

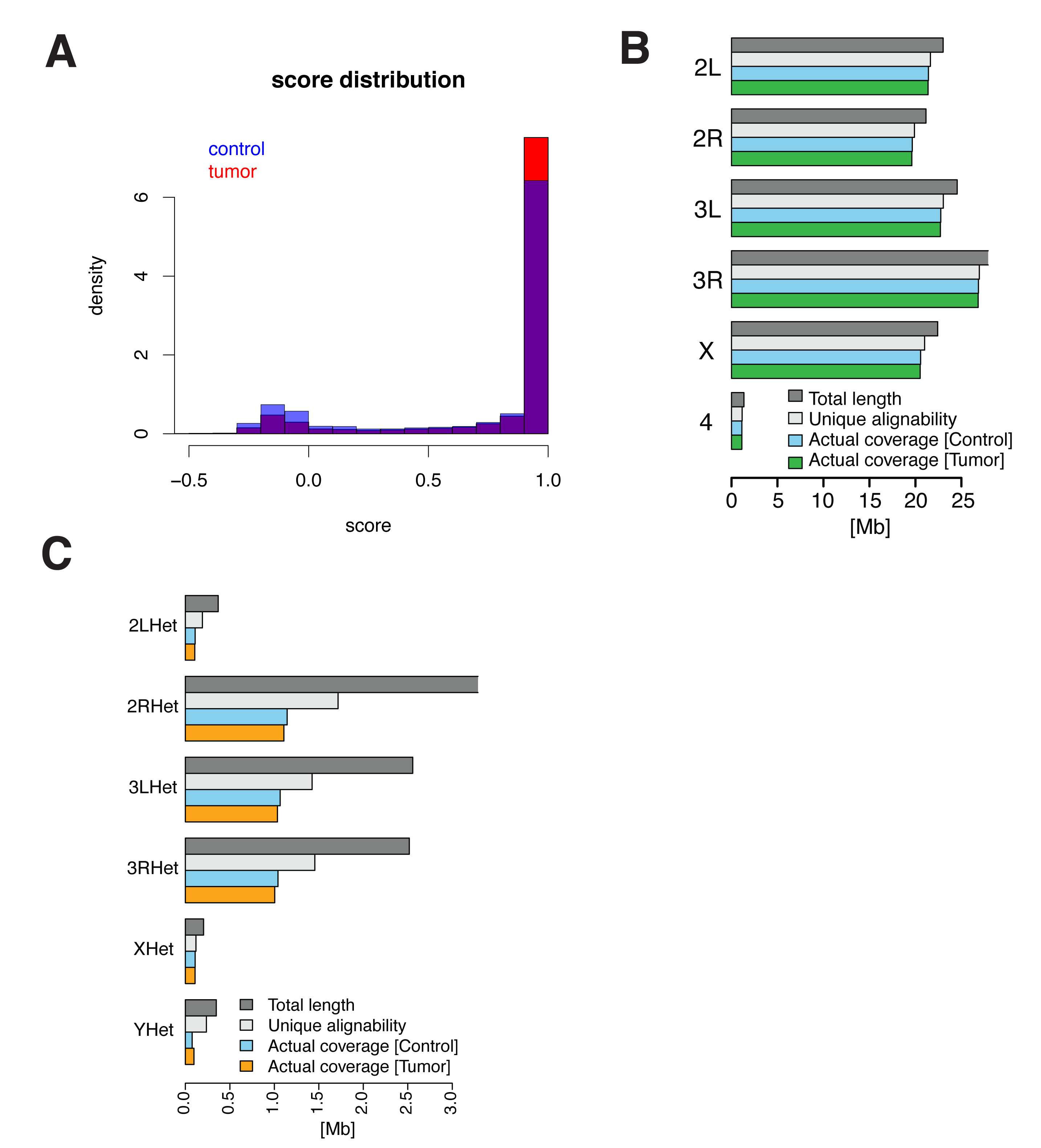

Supplement: Figure S4 — Assessing the characteristic uniqueness of the reference genome. (A) Bimodal distribution of the maximum score values obtained from reads pairs of the tumor and the control. The mode around zero corresponds to read pairs without sequence similarity derived from fragments longer than 287 bp as a minimum overlap of 13 was required (Methods). The mode around one corresponds to overlapping read pairs. Mismatches within overlapping regions can lead to score values close to and smaller than one. The probability mass distribution between the two modes is different between the tumor and the control: the first mode is less pronounced within the tumor, where more probability mass localizes at one. This can be explained by the fact that the tumor derived fragments are on average shorter than fragments from the control sample, resulting in a larger fraction of overlapping read pairs ultimately contributing to the second mode (Fig. S2D–E). (B, C) Assessment of the read coverage within euchromatic (B) or heterochromatic (C) regions obtained from each sequencing experiment. Bars indicate the total length of the chromosomes, the unique alignability resulting from = 30, and the length of the genomic region covered by concordant or discordant reads, both in the control and the tumor. (TIF) [file pone.0087090.s004.tif]

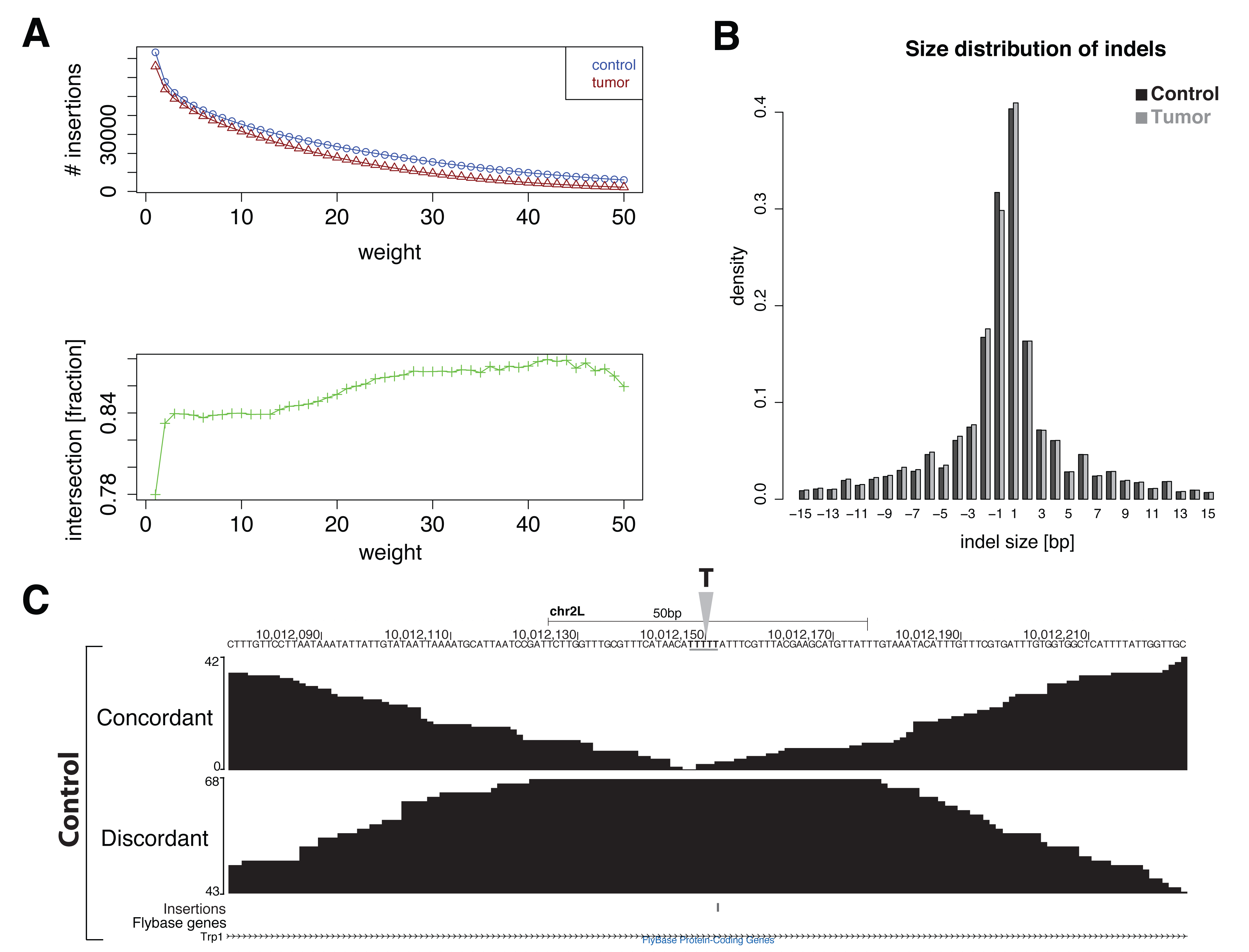

Supplement: Figure S5 — Characterization of the small insertions. (A) The top panel represents the total number of identified small insertions as a function of the minimum required weight (), i.e. the number of read pairs supporting the same event. The lower panel shows the fraction of small insertions present in both samples as a function of the . (B) Size distribution of recognized insertions/deletions. Positive and negative integers correspond to small insertions and deletions, respectively, of corresponding size. (C) Exemplary browser view of a T insertion within a simple T repeat of length 5. The same insertion was detected within the tumor and the control. (TIF) [file pone.0087090.s005.tif]

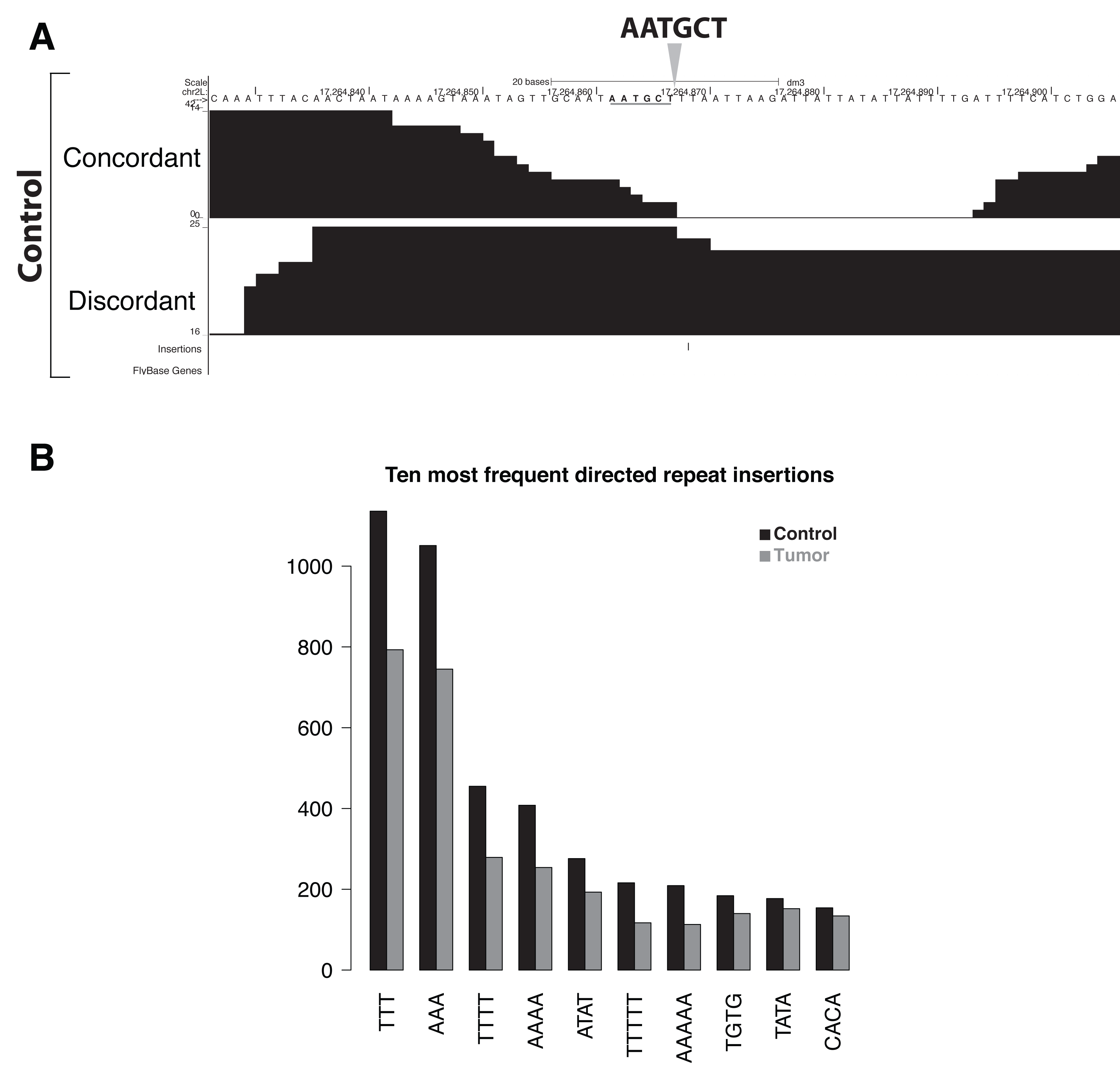

Supplement: Figure S6 — Small insertions can result from the conservative transposition. (A) Exemplary browser view of an insertion of length 5 resulting in a non-simple directed repeat. The same insertion was detected within the tumor and the control. (B) The 10 most frequent insertions of minimum length 3 resulting in the formation of a directed repeat in the control and the tumor. (TIF) [file pone.0087090.s006.tif]

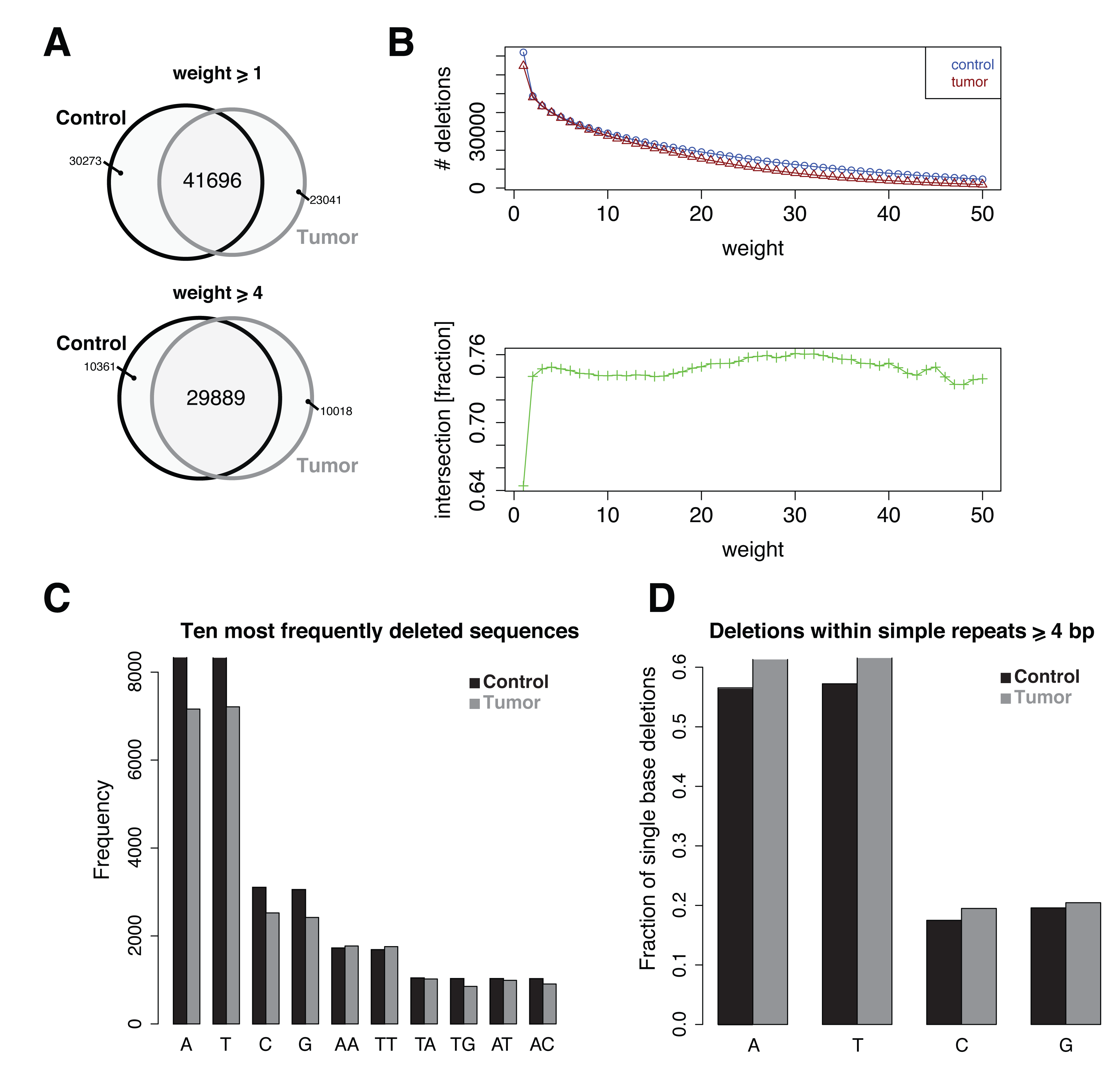

Supplement: Figure S7 — Characterization of the deletions. (A) Venn diagram representing the number of deletions identified within the tumor and the control. Upper panel corresponds to deletions of weight , lower panel shows deletions of weight . (B) The top panel represents the total number of identified deletions as a function of the minimum required weight (). The lower panel shows the fraction of deletions present in both samples as a function of the . (C) Frequency and DNA sequence of the 10 most commonly deleted sequences in the tumor and the control genomes. (D) The fraction of single base deletions within simple repeats consisting of the same base. Simple repeats of minimum length of 4 bp were considered for the analysis. The fraction is computed with respect to all corresponding single base deletions. (TIF) [file pone.0087090.s007.tif]

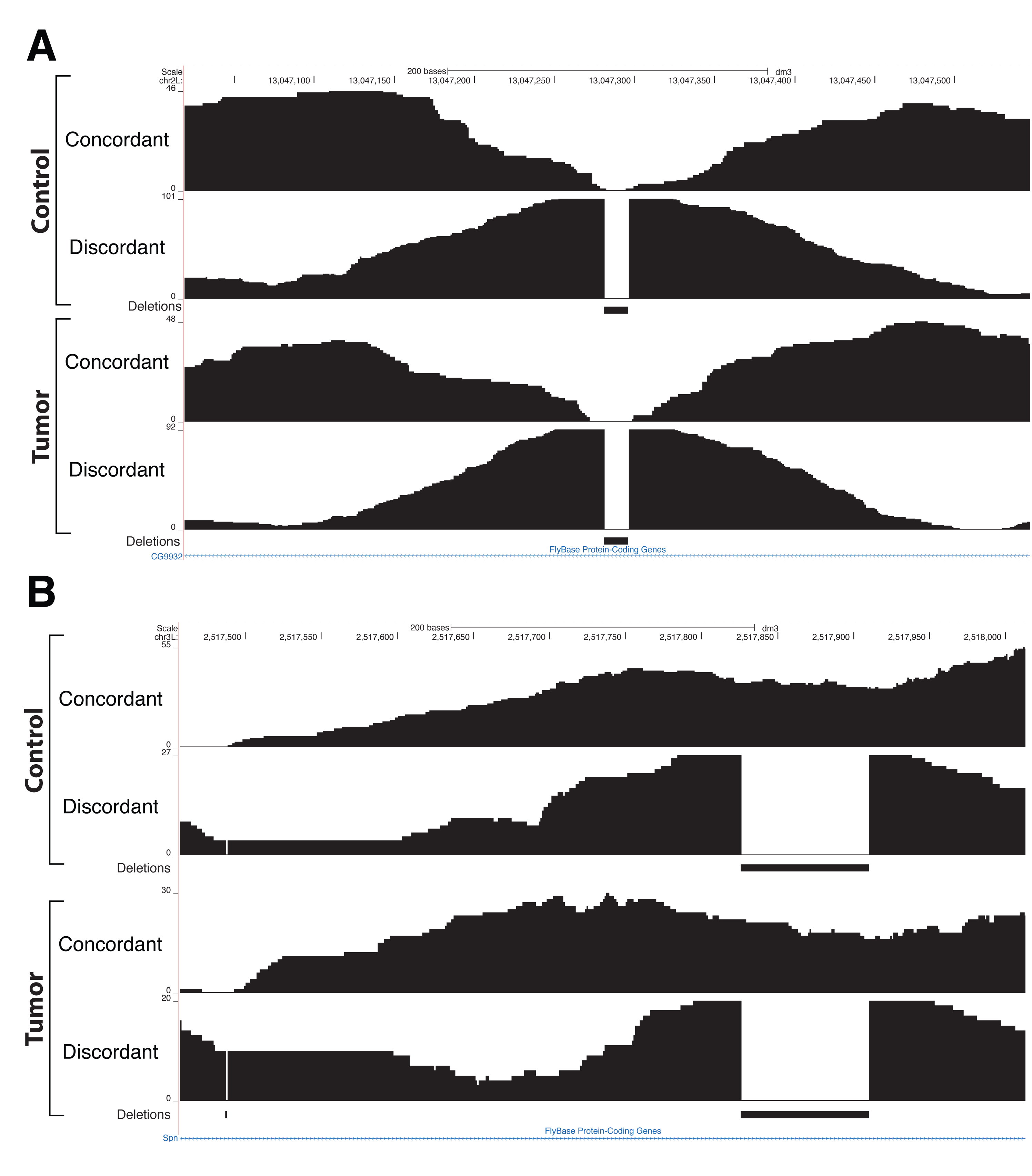

Supplement: Figure S8 — Examples of homozygous and heterozygous deletions. (A) Genome browser view of a homozygous deletion identified within both samples. In both cases the concordant coverage decreases towards zero. (B) Genome browser view of a heterozygous deletion identified within both samples. In both cases the concordant coverage shows a decreases across the affected region. (TIF) [file pone.0087090.s008.tif]

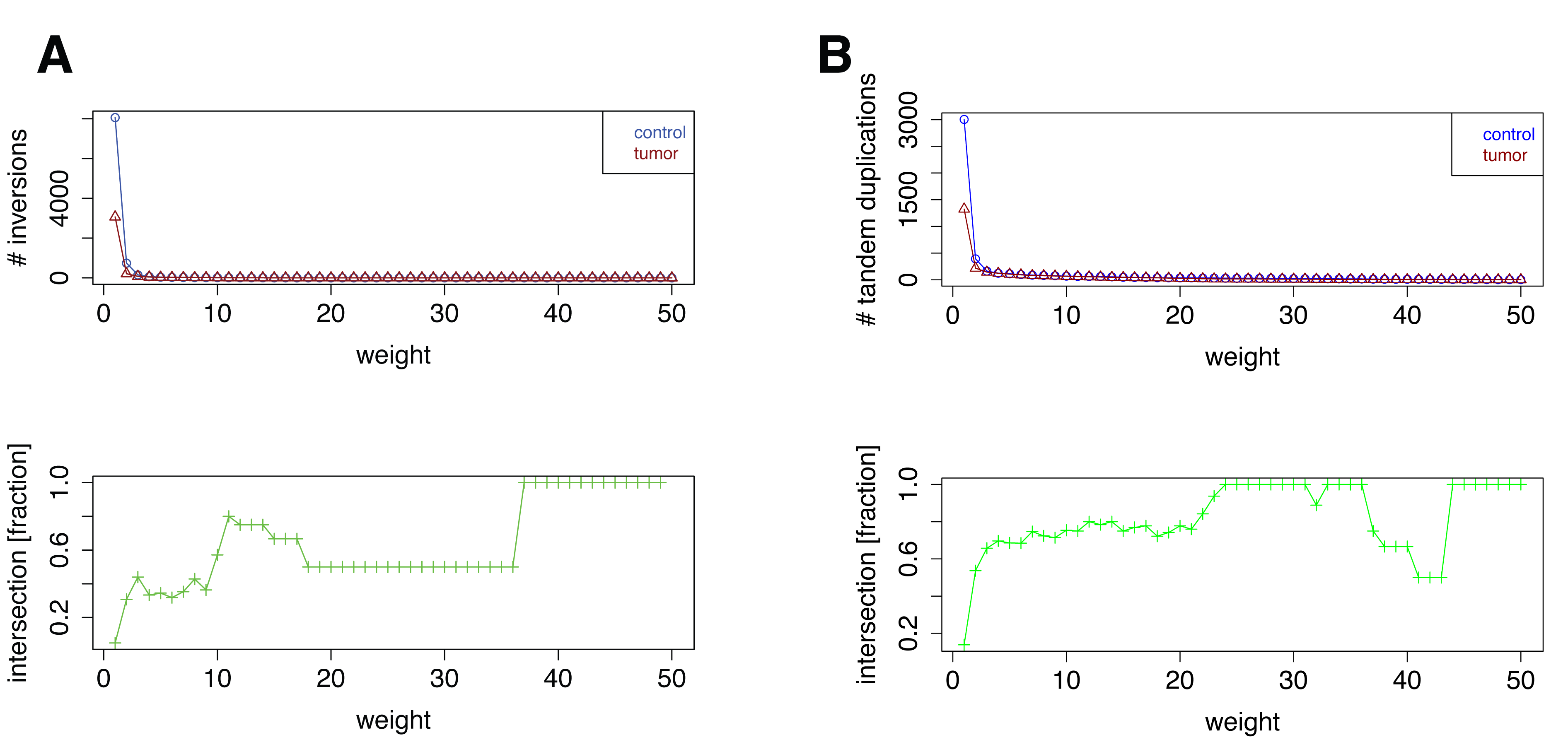

Supplement: Figure S9 — Characterization of inversions and tandem duplications. The top panel represents the total number of identified inversions (A) and tandem duplications (B) as a function of the . The lower panel shows the fraction of the according SV present in both samples as a function of the . In both cases 10 base tolerance per breakpoint was considered, as the formation mechanism of such events may involve repetitive sequences [38], leading to ambiguity in the breakpoint detection caused by multiple optimal alignments. The large variance in the lower panel of (A) is caused by the low absolute number of inversions, e.g. for a weight we observe 10 and 5 inversions for control and tumor, respectively. (TIF) [file pone.0087090.s009.tif]

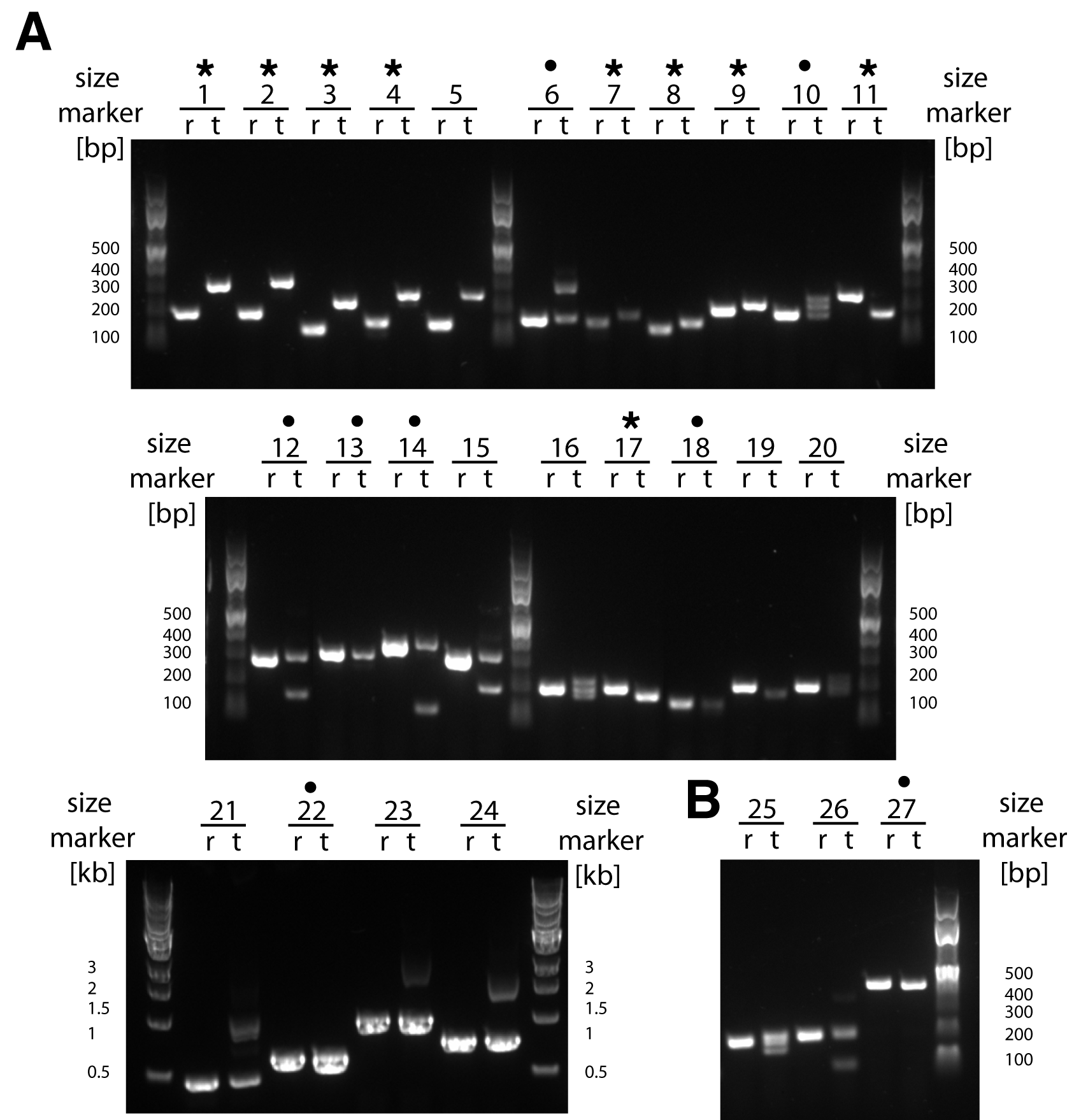

Supplement: Figure S10 — PCR-based validation of small insertions, deletions and tandem duplications. (A) PCR primers were designed to test different homozygous and heterozygous SVs. The selected SVs and their characteristics are described in Table S3. The numbering of the lanes corresponds to the SV IDs (Table S3). PCR was performed with genomic DNA of a Tumor (t) (obtained from the same ph RNAi strain as the tumors used for sequencing) and the reference strain (r) used to generate the D. melanogaster reference genome. In case of heterozygous events two PCR products are expected. (B) Same as (A), where the SVs indicated in Figure 4C have been tested. indicates SVs further confirmed using Sanger sequencing (Table S5); indicates heterozygous SVs that were also analyzed in the parental strains. (TIF) [file pone.0087090.s010.tif]

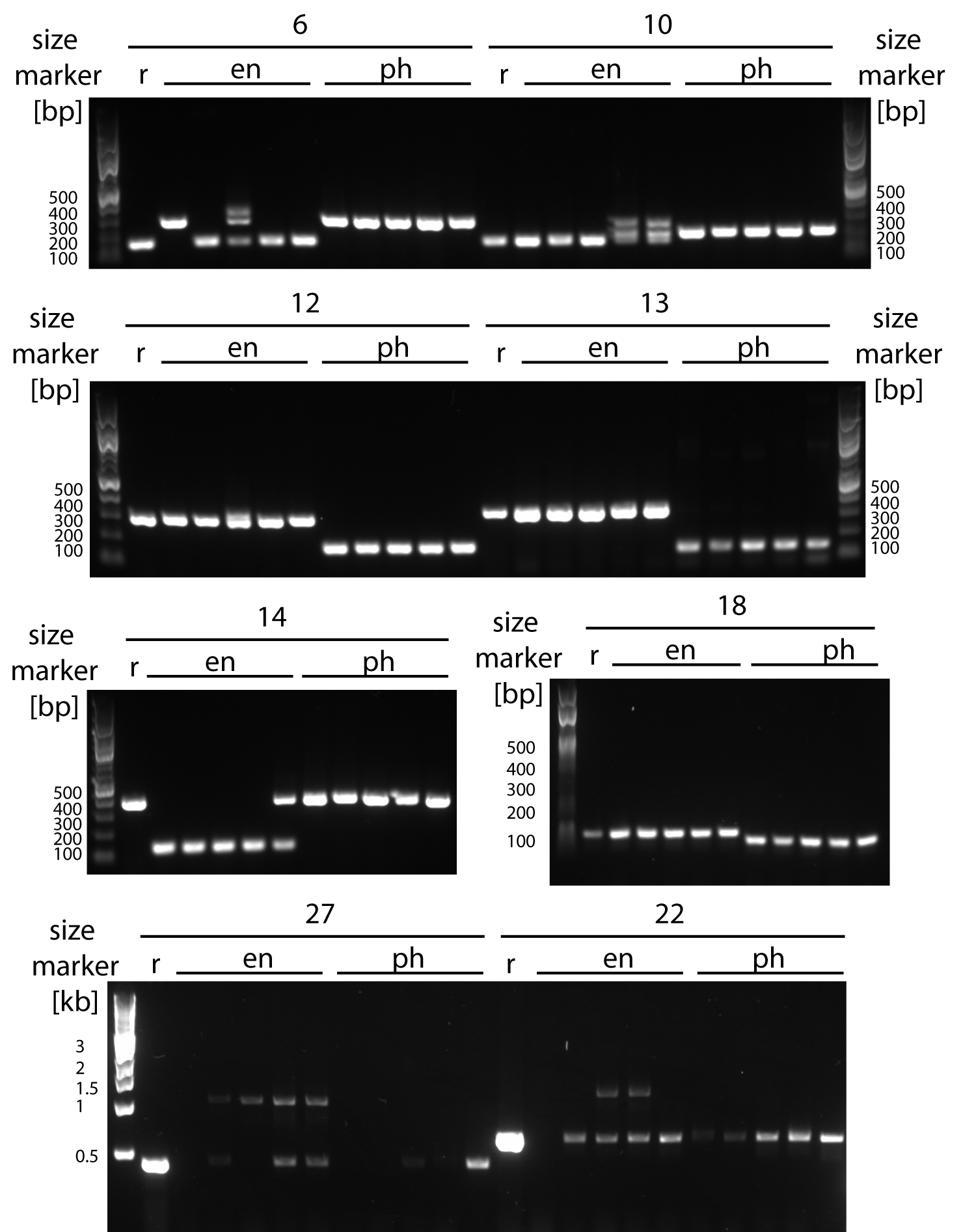

Supplement: Figure S11 — PCR-based validation of selected SVs in the parental strains. SVs that failed to be detected in Figure S10 (lanes 13, 18, 22 and 27) in addition to selected heterozygous events (Figure S10) were further tested in the parental strains. PCR was performed with genomic DNA of five individuals of each parental strain, indicated as en (engrailed) and ph (polyhomeotic, see Methods for details) and with genomic DNA from the reference strain (r). The observed size of the tandem duplication (27, corresponding to event II in Figure 4C in the main text) is approximately three times the size of a single duplication event. Since single and multiple tandem duplications cannot be distinguished based on the signature on the reference genome, this size increase possibly indicates three consecutive duplication events. (TIF) [file pone.0087090.s011.tif]
