# Supplementary material for: A Deterministic Analysis of Genome Integrity during Neoplastic Growth in Drosophila
Source: PLoS One. 2014 Feb 6;9(2):e87090. doi: 10.1371/journal.pone.0087090 (PMC3916295; doi:10.1371/journal.pone.0087090)
Supplement: Table S1 — Effects on coding sequences caused by small insertions of weight (w) specifically found within the tumor or the control. Indicated are genomic coordinates of the insertion (chr, pos), gene strand (strand), discordant coverage (w) as well as concordant coverage at the breakpoint (conc. coverage), location within the coding sequence (CDS location), Flybase gene ID (Gene ID) and the consequence of the insertion on the amino acid sequence (Consequence). (PDF) [file pone.0087090.s012.pdf]

Supporting Information for: "A deterministic analysis of genome integrity during neoplastic growth in *Drosophila*" Table S1

Cem Sievers[1], Federico Comoglio[1], Makiko Seimiya[1], Gunter Merdes[1,*] and Renato Paro[1,2,*]

[1]Department of Biosystems Science and Engineering, Swiss Federal Institute of Technology Zurich, Mattenstrasse 26, 4058 Basel, Switzerland

[2]Faculty of Science, University of Basel, Klingelbergstrasse 50, 4056 Basel, Switzerland

July 12, 2013

no.	chr	pos	strand	w	conc. coverage	CDS location	Gene ID	Consequence
				**Tumor specific insertions**				
1	2L	4123376	+	4	26	1426	FBgn0014033	nonsynonymous
2	2L	13272831	+	5	2	1286	FBgn0032497	nonsynonymous
3	2L	13272831	+	5	2	1517	FBgn0032497	nonsynonymous
4	2L	13272831	+	5	2	1286	FBgn0032497	nonsynonymous
5	2L	13272831	+	5	2	1286	FBgn0032497	nonsynonymous
6	2L	13272831	+	5	2	1286	FBgn0032497	nonsynonymous
7	2L	13272831	+	5	2	1517	FBgn0032497	nonsynonymous
8	2L	13272831	+	5	2	1517	FBgn0032497	nonsynonymous
9	2L	13272831	+	5	2	1517	FBgn0032497	nonsynonymous
10	2L	13272831	+	5	2	1517	FBgn0032497	nonsynonymous
11	2L	16658075	+	5	11	2492	FBgn0259735	nonsynonymous
12	2L	16658075	+	5	11	1586	FBgn0259735	nonsynonymous
13	2L	16658075	+	5	11	539	FBgn0259735	nonsynonymous
14	2L	12498706	-	6	26	64	FBgn0259176	nonsynonymous
15	2L	16728782	-	6	17	283	FBgn0051782	frameshift
16	2L	16728782	-	6	17	283	FBgn0051782	frameshift
17	2R	7613207	+	5	81	1703	FBgn0033649	nonsynonymous
18	2R	12957513	-	13	20	1195	FBgn0050460	nonsynonymous
19	2R	12957513	-	13	20	1549	FBgn0050460	nonsynonymous
20	2R	12957513	-	13	20	1549	FBgn0050460	nonsynonymous
21	2R	12957513	-	13	20	2593	FBgn0050460	nonsynonymous
22	2R	12957513	-	13	20	2386	FBgn0050460	nonsynonymous
23	2R	12957513	-	13	20	2122	FBgn0050460	nonsynonymous
24	2R	12957513	-	13	20	1195	FBgn0050460	nonsynonymous
25	2R	13565884	-	5	19	26	FBgn0016059	nonsynonymous
26	2R	13565884	-	5	19	26	FBgn0016059	nonsynonymous
27	2R	13565884	-	5	19	26	FBgn0016059	nonsynonymous
28	2R	19301143	-	4	4	555	FBgn0034860	nonsynonymous
29	3L	9830341	-	5	6	1303	FBgn0015618	nonsynonymous
30	3L	16918205	-	5	11	880	FBgn0036677	nonsynonymous
31	3R	13956507	+	14	7	1865	FBgn0003499	nonsynonymous
32	3R	13956507	+	14	7	1025	FBgn0003499	nonsynonymous
33	3R	22457957	+	4	10	305	FBgn0039441	nonsynonymous
34	3R	25705079	+	5	12	986	FBgn0051038	nonsynonymous
35	3R	25705079	+	5	12	986	FBgn0051038	nonsynonymous
36	3R	25705079	+	5	12	986	FBgn0051038	nonsynonymous
37	3R	25705079	+	5	12	986	FBgn0051038	nonsynonymous
38	3R	25705079	+	5	12	986	FBgn0051038	nonsynonymous
39	3R	10130741	-	14	36	2615	FBgn0003567	nonsynonymous
40	3R	10130741	-	14	36	2615	FBgn0003567	nonsynonymous
41	3R	17168761	-	14	2	109	FBgn0038881	nonsynonymous
42	X	5847916	+	5	1	962	FBgn0029827	nonsynonymous
43	X	12338985	+	9	34	1755	FBgn0259680	nonsynonymous
44	X	5892506	-	13	31	1107	FBgn0027546	frameshift
				**Control specific insertions**				

Continued on next page

no.	chr	pos	strand	w	conc. coverage	CDS location	Gene ID	Consequence
1	2L	1606970	+	4	4	3304	FBgn0261509	nonsynonymous
2	2L	4815259	+	14	68	511	FBgn0031630	frameshift
3	2L	4865387	+	12	29	85	FBgn0051660	nonsynonymous
4	2L	4865387	+	12	29	85	FBgn0051660	nonsynonymous
5	2L	8175364	+	8	25	1236	FBgn0031993	nonsynonymous
6	2L	8175364	+	8	25	1236	FBgn0031993	nonsynonymous
7	2L	8175364	+	8	25	1236	FBgn0031993	nonsynonymous
8	2L	8427730	+	7	11	695	FBgn0027932	nonsynonymous
9	2L	8427730	+	7	11	695	FBgn0027932	nonsynonymous
10	2L	8427730	+	7	11	695	FBgn0027932	nonsynonymous
11	2L	8427730	+	7	11	695	FBgn0027932	nonsynonymous
12	2L	10055698	+	5	19	849	FBgn0053302	nonsynonymous
13	2L	10055698	+	5	19	849	FBgn0053302	nonsynonymous
14	2L	561604	-	6	30	1578	FBgn0031264	nonsynonymous
15	2L	7499027	-	7	38	2386	FBgn0085403	nonsynonymous
16	2L	7499027	-	7	38	2551	FBgn0085403	nonsynonymous
17	2L	7499027	-	7	38	2629	FBgn0085403	nonsynonymous
18	2L	7499027	-	7	38	2800	FBgn0085403	nonsynonymous
19	2L	10068904	-	8	8	2770	FBgn0032180	not translated
20	2L	12537098	-	4	19	2278	FBgn0259176	nonsynonymous
21	2L	12537098	-	4	19	1951	FBgn0259176	nonsynonymous
22	2L	12537098	-	4	19	1951	FBgn0259176	nonsynonymous
23	2L	16339684	-	13	1	568	FBgn0259151	nonsynonymous
24	2L	18131278	-	6	51	3930	FBgn0001301	nonsynonymous
25	2L	18533680	-	6	51	4011	FBgn0085370	nonsynonymous
26	2L	18533680	-	6	51	3879	FBgn0085370	nonsynonymous
27	2L	19756028	-	4	41	493	FBgn0032817	nonsynonymous
28	2R	2123376	+	12	5	1720	FBgn0033073	nonsynonymous
29	2R	2123376	+	12	5	1720	FBgn0033073	nonsynonymous
30	2R	2839661	+	5	3	1354	FBgn0053349	frameshift
31	2R	6498338	+	13	29	599	FBgn0004399	nonsynonymous
32	2R	6498338	+	13	29	599	FBgn0004399	nonsynonymous
33	2R	6498338	+	13	29	599	FBgn0004399	nonsynonymous
34	2R	6498338	+	13	29	599	FBgn0004399	nonsynonymous
35	2R	9926702	+	4	0	1184	FBgn0002643	nonsynonymous
36	2R	9926702	+	4	0	1184	FBgn0002643	nonsynonymous
37	2R	9926702	+	4	0	500	FBgn0002643	nonsynonymous
38	2R	9926702	+	4	0	1184	FBgn0002643	nonsynonymous
39	2R	9926702	+	4	0	1184	FBgn0002643	nonsynonymous
40	2R	9926702	+	4	0	500	FBgn0002643	nonsynonymous
41	2R	9927618	+	13	1	2100	FBgn0002643	nonsynonymous
42	2R	9927618	+	13	1	2100	FBgn0002643	nonsynonymous
43	2R	10393244	+	6	10	425	FBgn0000142	nonsynonymous
44	2R	10393244	+	6	10	425	FBgn0000142	nonsynonymous
45	2R	13708702	+	6	2	1442	FBgn0259211	nonsynonymous
46	2R	13708702	+	6	2	1442	FBgn0259211	nonsynonymous
47	2R	13708702	+	6	2	1442	FBgn0259211	nonsynonymous
48	2R	13708702	+	6	2	1442	FBgn0259211	nonsynonymous
49	2R	13708702	+	6	2	605	FBgn0259211	nonsynonymous
50	2R	19234281	+	7	20	91	FBgn0034846	nonsynonymous
51	2R	19344868	+	4	10	856	FBgn0034862	nonsynonymous
52	2R	4999721	-	8	39	1594	FBgn0033379	nonsynonymous
53	2R	9809208	-	6	42	274	FBgn0013733	nonsynonymous
54	2R	9809208	-	6	42	274	FBgn0013733	nonsynonymous
55	2R	9809208	-	6	42	274	FBgn0013733	nonsynonymous
56	2R	9809208	-	6	42	274	FBgn0013733	nonsynonymous
57	2R	9809208	-	6	42	274	FBgn0013733	nonsynonymous
58	2R	10563402	-	14	48	2192	FBgn0050480	nonsynonymous
59	2R	12072760	-	8	54	671	FBgn0034086	nonsynonymous
60	2R	12754881	-	15	40	22	FBgn0014870	nonsynonymous
61	2R	12754881	-	15	40	22	FBgn0014870	nonsynonymous
62	2R	14064130	-	13	41	1756	FBgn0034313	nonsynonymous
63	2R	16717771	-	11	13	105	FBgn0016984	nonsynonymous
64	2R	18232414	-	6	0	499	FBgn0034720	nonsynonymous
65	2R	18232414	-	6	0	112	FBgn0034720	nonsynonymous
66	2R	18232414	-	6	0	112	FBgn0034720	nonsynonymous
67	2R	18232414	-	6	0	112	FBgn0034720	nonsynonymous
68	3L	1240112	+	8	36	875	FBgn0035192	nonsynonymous
69	3L	1822468	+	15	0	576	FBgn0027790	nonsynonymous
70	3L	1822468	+	15	0	576	FBgn0027790	nonsynonymous
71	3L	4418395	+	8	24	274	FBgn0035542	nonsynonymous
72	3L	4418395	+	8	24	274	FBgn0035542	nonsynonymous
73	3L	4418395	+	8	24	274	FBgn0035542	nonsynonymous
74	3L	4418395	+	8	24	274	FBgn0035542	nonsynonymous
75	3L	5875817	+	8	39	311	FBgn0035643	nonsynonymous
76	3L	6073466	+	7	11	1694	FBgn0035676	nonsynonymous
77	3L	8406175	+	7	70	1	FBgn0010431	frameshift
78	3L	9605816	+	8	28	4861	FBgn0036017	nonsynonymous
79	3L	10625324	+	6	0	757	FBgn0001179	nonsynonymous
80	3L	10625324	+	6	0	757	FBgn0001179	nonsynonymous
81	3L	10863287	+	5	18	365	FBgn0026160	nonsynonymous

Continued on next page

no.	chr	pos	strand	w	conc. coverage	CDS location	Gene ID	Consequence
82	3L	10863287	+	5	18	212	FBgn0026160	nonsynonymous
83	3L	10863287	+	5	18	134	FBgn0026160	nonsynonymous
84	3L	10863287	+	5	18	254	FBgn0026160	nonsynonymous
85	3L	16126942	+	5	45	1106	FBgn0036574	nonsynonymous
86	3L	16126942	+	5	45	1274	FBgn0036574	nonsynonymous
87	3L	20314180	+	7	0	40	FBgn0036969	nonsynonymous
88	3L	1614435	-	5	4	1141	FBgn0035241	nonsynonymous
89	3L	6724446	-	10	0	6523	FBgn0052394	nonsynonymous
90	3L	6725070	-	4	39	5899	FBgn0052394	nonsynonymous
91	3L	10874714	-	8	26	261	FBgn0036106	nonsynonymous
92	3L	11605144	-	5	6	8029	FBgn0036181	nonsynonymous
93	3L	11605144	-	5	6	8029	FBgn0036181	nonsynonymous
94	3L	11605144	-	5	6	8029	FBgn0036181	not translated
95	3L	11957416	-	7	5	436	FBgn0028573	nonsynonymous
96	3L	18051679	-	4	15	1006	FBgn0000568	nonsynonymous
97	3L	19457494	-	7	5	227	FBgn0036870	nonsynonymous
98	3R	11686678	+	12	5	609	FBgn0038377	nonsynonymous
99	3R	11686678	+	12	5	441	FBgn0038377	nonsynonymous
100	3R	12914780	+	13	28	600	FBgn0038478	nonsynonymous
101	3R	13241887	+	4	51	420	FBgn0038508	nonsynonymous
102	3R	14994010	+	4	9	254	FBgn0010768	nonsynonymous
103	3R	20840647	+	4	13	2108	FBgn0039257	nonsynonymous
104	3R	21083465	+	8	44	7343	FBgn0039302	nonsynonymous
105	3R	21488552	+	11	49	269	FBgn0039352	nonsynonymous
106	3R	23166484	+	10	14	141	FBgn0260487	nonsynonymous
107	3R	25816088	+	6	18	4007	FBgn0039728	nonsynonymous
108	3R	25878899	+	7	4	379	FBgn0039743	nonsynonymous
109	3R	26600166	+	5	0	719	FBgn0004606	nonsynonymous
110	3R	1001248	-	12	21	4073	FBgn0259212	nonsynonymous
111	3R	1001248	-	12	21	4580	FBgn0259212	nonsynonymous
112	3R	4662727	-	5	37	3925	FBgn0003177	nonsynonymous
113	3R	4844180	-	5	49	1225	FBgn0015014	nonsynonymous
114	3R	5070096	-	11	83	1126	FBgn0260935	nonsynonymous
115	3R	5070096	-	11	83	1126	FBgn0260935	nonsynonymous
116	3R	7991171	-	5	26	102	FBgn0010218	nonsynonymous
117	3R	7991171	-	5	26	102	FBgn0010218	nonsynonymous
118	3R	8817865	-	4	88	750	FBgn0041710	frameshift
119	3R	10355217	-	6	21	297	FBgn0038237	nonsynonymous
120	3R	17544783	-	4	21	280	FBgn0051176	nonsynonymous
121	3R	17911474	-	4	5	205	FBgn0038930	nonsynonymous
122	3R	17911474	-	4	5	205	FBgn0038930	nonsynonymous
123	X	3185386	+	7	0	135	FBgn0000479	nonsynonymous
124	X	5803091	+	11	0	458	FBgn0029822	nonsynonymous
125	X	5803091	+	11	0	458	FBgn0029822	nonsynonymous
126	X	5803092	+	4	0	459	FBgn0029822	nonsynonymous
127	X	5803092	+	4	0	459	FBgn0029822	nonsynonymous
128	X	5844435	+	4	46	27	FBgn0052755	nonsynonymous
129	X	8373585	+	11	5	107	FBgn0000359	nonsynonymous
130	X	9504258	+	8	13	860	FBgn0030144	nonsynonymous
131	X	17100970	+	18	1	463	FBgn0052564	nonsynonymous
132	X	4138811	-	6	0	895	FBgn0052774	not translated
133	X	4138811	-	6	0	895	FBgn0052774	nonsynonymous
134	X	6378991	-	4	0	109	FBgn0259242	nonsynonymous
135	X	11822591	-	33	11	1570	FBgn0030358	nonsynonymous
136	X	12685720	-	8	4	4556	FBgn0259171	nonsynonymous
137	X	14684787	-	17	4	5731	FBgn0003301	nonsynonymous
138	X	21273590	-	4	27	155	FBgn0031176	nonsynonymous
139	X	21273590	-	4	27	155	FBgn0031176	not translated

End of the table
