# Supplementary material for: A Deterministic Analysis of Genome Integrity during Neoplastic Growth in Drosophila
Source: PLoS One. 2014 Feb 6;9(2):e87090. doi: 10.1371/journal.pone.0087090 (PMC3916295; doi:10.1371/journal.pone.0087090)
Supplement: Table S2 — Effects on coding sequences caused by deletions of weight (w) specifically found within the tumor or the control. Indicated are genomic coordinates of the deletion (chr, start, end), gene strand (strand), discordant coverage (w) as well as concordant coverage at the breakpoints (conc. cov. 1, conc. cov. 2), location within the coding sequence (CDS start, CDS end), Flybase gene ID (Gene ID) and the consequence of the deletion on the amino acid sequence (Consequence). (PDF) [file pone.0087090.s013.pdf]

Supporting Information for: "A deterministic analysis of genome integrity during neoplastic growth in *Drosophila*" Table S2

Cem Sievers[1], Federico Comoglio[1], Makiko Seimiya[1], Gunter Merdes[1,*] and Renato Paro[1,2,*]

[1]Department of Biosystems Science and Engineering, Swiss Federal Institute of Technology Zurich, Mattenstrasse 26, 4058 Basel, Switzerland

[2]Faculty of Science, University of Basel, Klingelbergstrasse 50, 4056 Basel, Switzerland

July 12, 2013

no.	chr	start	end	strand	w	conc. cov. 1	conc. cov. 2	CDS start	CDS end	Gene ID	Consequence
**Tumor specific deletions**											
1	2L	8184286	8184295	+	16	0	0	5412	5421	FBgn0031993	nonsynonymous
2	2L	8184286	8184295	+	16	0	0	5412	5421	FBgn0031993	nonsynonymous
3	2L	8184286	8184295	+	16	0	0	5190	5199	FBgn0031993	nonsynonymous
4	2L	10648293	10648338	+	7	11	11	1795	1840	FBgn0032275	nonsynonymous
5	2L	13777549	13777558	+	7	13	13	341	350	FBgn0051845	nonsynonymous
6	2L	18991547	18991553	+	16	25	26	473	479	FBgn0032734	nonsynonymous
7	2L	20771772	20771781	+	4	1	1	665	674	FBgn0000251	nonsynonymous
8	2L	20771772	20771781	+	4	1	1	665	674	FBgn0000251	nonsynonymous
9	2L	1499064	1499073	-	14	9	9	799	808	FBgn0031343	nonsynonymous
10	2L	1499064	1499073	-	14	9	9	916	925	FBgn0031343	nonsynonymous
11	2L	1499064	1499073	-	14	9	9	916	925	FBgn0031343	nonsynonymous
12	2L	1499064	1499073	-	14	9	9	799	808	FBgn0031343	nonsynonymous
13	2L	1499064	1499073	-	14	9	9	1126	1135	FBgn0031343	nonsynonymous
14	2L	1499064	1499073	-	14	9	9	1009	1018	FBgn0031343	nonsynonymous
15	2L	8447493	8447496	-	4	55	54	1694	1697	FBgn0027780	nonsynonymous
16	2L	10831826	10831841	-	16	4	6	87	102	FBgn0011676	nonsynonymous
17	2L	10831826	10831841	-	16	4	6	87	102	FBgn0011676	nonsynonymous
18	2L	10831826	10831841	-	16	4	6	87	102	FBgn0011676	nonsynonymous
19	2L	10831826	10831841	-	16	4	6	87	102	FBgn0011676	nonsynonymous
20	2L	10831826	10831841	-	16	4	6	87	102	FBgn0011676	nonsynonymous
21	2L	10831826	10831841	-	16	4	6	87	102	FBgn0011676	nonsynonymous
22	2L	10831826	10831841	-	16	4	6	87	102	FBgn0011676	nonsynonymous
23	2L	10831826	10831841	-	16	4	6	87	102	FBgn0011676	nonsynonymous
24	2L	10831826	10831841	-	16	4	6	87	102	FBgn0011676	nonsynonymous
25	2L	10831826	10831841	-	16	4	6	87	102	FBgn0011676	nonsynonymous
26	2L	11821212	11821218	-	4	9	8	67	73	FBgn0259225	nonsynonymous
27	2R	7096785	7096788	+	20	2	2	1124	1127	FBgn0003396	nonsynonymous
28	2R	7096785	7096788	+	20	2	2	989	992	FBgn0003396	nonsynonymous
29	2R	7096785	7096788	+	20	2	2	989	992	FBgn0003396	nonsynonymous
30	2R	7096785	7096788	+	20	2	2	989	992	FBgn0003396	nonsynonymous
31	2R	8357014	8357026	+	24	12	12	695	707	FBgn0033741	nonsynonymous
32	2R	9848730	9848744	+	19	0	0	32	46	FBgn0013770	frameshift
33	2R	17537882	17537888	+	4	12	12	152	158	FBgn0034644	nonsynonymous
34	2R	17537885	17537891	+	6	12	12	155	161	FBgn0034644	nonsynonymous
35	2R	18708967	18709021	+	4	0	5	1319	1373	FBgn0050271	nonsynonymous
36	2R	18708967	18709021	+	4	0	5	1277	1331	FBgn0050271	nonsynonymous
37	2R	19956841	19956844	+	8	43	42	119	122	FBgn0034971	nonsynonymous
38	2R	19956841	19956844	+	8	43	42	119	122	FBgn0034971	nonsynonymous
39	2R	5604925	5604937	-	16	3	2	1754	1766	FBgn0259678	nonsynonymous
40	2R	7865240	7865249	-	4	61	66	1151	1160	FBgn0050036	nonsynonymous
41	2R	11863083	11863473	-	6	5	0	9213	9603	FBgn0013988	nonsynonymous
42	2R	11863083	11863473	-	6	5	0	9213	9603	FBgn0013988	nonsynonymous
43	2R	11863083	11863473	-	6	5	0	9213	9603	FBgn0013988	nonsynonymous
44	2R	11863083	11863473	-	6	5	0	9213	9603	FBgn0013988	nonsynonymous
45	2R	11863083	11863473	-	6	5	0	9213	9603	FBgn0013988	nonsynonymous
46	2R	11863083	11863473	-	6	5	0	9213	9603	FBgn0013988	nonsynonymous

Continued on next page

no.	chr	start	end	strand	w	conc. cov. 1	conc. cov. 2	CDS start	CDS end	Gene ID	Consequence
47	2R	11863083	11863473	-	6	5	0	9213	9603	FBgn0013988	nonsynonymous
48	3L	547978	547981	+	11	21	21	200	203	FBgn0035142	nonsynonymous
49	3L	547978	547981	+	11	21	21	200	203	FBgn0035142	nonsynonymous
50	3L	12966418	12966429	+	4	38	39	793	804	FBgn0036328	frameshift
51	3L	15547518	15547536	+	22	0	0	145	163	FBgn0087035	nonsynonymous
52	3L	15547518	15547536	+	22	0	0	154	172	FBgn0087035	nonsynonymous
53	3L	1632311	1632317	-	4	4	4	502	508	FBgn0013342	nonsynonymous
54	3L	1632311	1632317	-	4	4	4	529	535	FBgn0013342	nonsynonymous
55	3L	1632311	1632317	-	4	4	4	529	535	FBgn0013342	nonsense
56	3L	1632311	1632317	-	4	4	4	502	508	FBgn0013342	nonsynonymous
57	3L	3113605	3113608	-	21	25	24	450	453	FBgn0035399	nonsynonymous
58	3L	3113605	3113608	-	21	25	24	450	453	FBgn0035399	nonsynonymous
59	3L	8428759	8428768	-	5	59	61	2041	2050	FBgn0035895	nonsynonymous
60	3L	8428759	8428768	-	5	59	61	2095	2104	FBgn0035895	nonsynonymous
61	3L	14744702	14744705	-	12	1	0	1303	1306	FBgn0013263	nonsynonymous
62	3L	14744702	14744705	-	12	1	0	1303	1306	FBgn0013263	nonsynonymous
63	3L	14744702	14744705	-	12	1	0	1303	1306	FBgn0013263	nonsynonymous
64	3L	16359690	16359714	-	7	5	6	695	719	FBgn0036619	nonsynonymous
65	3L	16918216	16918234	-	5	11	10	851	869	FBgn0036677	nonsynonymous
66	3L	19251137	19251155	-	5	75	75	1450	1468	FBgn0026630	nonsynonymous
67	3L	19251137	19251155	-	5	75	75	1450	1468	FBgn0026630	nonsynonymous
68	3L	19251137	19251155	-	5	75	75	1450	1468	FBgn0026630	nonsynonymous
69	3L	19457482	19457494	-	4	0	1	227	239	FBgn0036870	nonsynonymous
70	3R	8244861	8244864	+	6	18	18	4531	4534	FBgn0082831	nonsynonymous
71	3R	9528527	9528553	+	4	47	47	1669	1695	FBgn0002937	frameshift
72	3R	11865828	11865834	+	4	3	3	1261	1267	FBgn0003117	nonsynonymous
73	3R	11865828	11865834	+	4	3	3	1417	1423	FBgn0003117	nonsynonymous
74	3R	17714948	17714951	+	4	20	20	1069	1072	FBgn0038917	nonsynonymous
75	3R	20064585	20064588	+	4	2	2	3608	3611	FBgn0043884	nonsynonymous
76	3R	20064585	20064588	+	4	2	2	3608	3611	FBgn0043884	nonsynonymous
77	3R	23533603	23533609	+	6	15	14	614	620	FBgn0039544	nonsynonymous
78	3R	23533603	23533609	+	6	15	14	614	620	FBgn0039544	nonsynonymous
79	3R	4660287	4660299	-	12	5	4	2743	2755	FBgn0003177	nonsynonymous
80	3R	4660287	4660299	-	12	5	4	2743	2755	FBgn0003177	nonsynonymous
81	3R	4660287	4660299	-	12	5	4	2743	2755	FBgn0003177	nonsynonymous
82	3R	4660287	4660299	-	12	5	4	3022	3034	FBgn0003177	nonsynonymous
83	3R	4660287	4660299	-	12	5	4	2977	2989	FBgn0003177	nonsynonymous
84	3R	4660287	4660299	-	12	5	4	4069	4081	FBgn0003177	nonsynonymous
85	3R	4660287	4660299	-	12	5	4	2977	2989	FBgn0003177	nonsynonymous
86	3R	8467809	8467818	-	10	19	20	9	18	FBgn0038070	nonsynonymous
87	3R	9807529	9807538	-	5	2	2	1110	1119	FBgn0086364	nonsynonymous
88	3R	10170736	10170739	-	8	13	13	154	157	FBgn0024321	nonsynonymous
89	3R	10170736	10170739	-	8	13	13	154	157	FBgn0024321	nonsynonymous
90	3R	13731107	13731110	-	6	4	4	961	964	FBgn0053547	nonsynonymous
91	3R	17677519	17677525	-	4	27	28	594	600	FBgn0015229	nonsynonymous
92	3R	18251484	18251490	-	15	18	19	2154	2160	FBgn0038967	nonsynonymous
93	3R	24439393	24439394	-	10	31	31	2355	2356	FBgn0039590	frameshift
94	X	5803361	5803367	+	12	0	0	728	734	FBgn0029822	nonsynonymous
95	X	5803361	5803367	+	12	0	0	728	734	FBgn0029822	nonsynonymous
96	X	9038661	9038670	+	6	24	25	1761	1770	FBgn0030091	nonsynonymous
97	X	9773019	9773043	+	14	1	3	420	444	FBgn0030157	nonsynonymous
98	X	10117254	10117257	+	7	0	0	1396	1399	FBgn0085437	nonsynonymous
99	X	10117254	10117257	+	7	0	0	97	100	FBgn0085437	nonsynonymous
100	X	7583167	7583170	-	10	3	4	859	862	FBgn0029504	nonsynonymous
101	X	7583167	7583170	-	10	3	4	859	862	FBgn0029504	nonsynonymous
102	X	8423504	8423507	-	12	4	2	52	55	FBgn0024943	nonsynonymous
103	X	9222309	9222315	-	14	2	2	1782	1788	FBgn0085478	nonsynonymous
104	X	9222309	9222315	-	14	2	2	1713	1719	FBgn0085478	nonsynonymous
105	X	10469937	10469940	-	14	0	0	280	283	FBgn0085443	nonsynonymous
106	X	11911522	11911525	-	13	26	25	382	385	FBgn0043001	nonsynonymous
**Control specific deletions**											
1	2L	1607052	1607058	+	4	12	13	3386	3392	FBgn0261509	nonsynonymous
2	2L	4885434	4885452	+	17	71	74	1737	1755	FBgn0031643	nonsynonymous
3	2L	5066964	5066973	+	4	33	34	563	572	FBgn0028572	nonsynonymous
4	2L	5066964	5066973	+	4	33	34	563	572	FBgn0028572	nonsynonymous
5	2L	5066964	5066973	+	4	33	34	563	572	FBgn0028572	nonsynonymous
6	2L	5066964	5066973	+	4	33	34	98	107	FBgn0028572	nonsynonymous
7	2L	5066964	5066973	+	4	33	34	98	107	FBgn0028572	nonsynonymous
8	2L	5066964	5066973	+	4	33	34	98	107	FBgn0028572	nonsynonymous
9	2L	5066964	5066973	+	4	33	34	98	107	FBgn0028572	nonsynonymous
10	2L	5066964	5066973	+	4	33	34	194	203	FBgn0028572	nonsynonymous
11	2L	5209088	5209094	+	7	120	124	1375	1381	FBgn0031688	nonsynonymous
12	2L	12129280	12129286	+	12	19	17	3716	3722	FBgn0032414	nonsynonymous
13	2L	16243395	16243422	+	6	101	96	4630	4657	FBgn0028859	nonsynonymous
14	2L	16559248	16559254	+	10	2	3	1120	1126	FBgn0259735	nonsynonymous
15	2L	16559248	16559254	+	10	2	3	1120	1126	FBgn0259735	nonsynonymous
16	2L	16813791	16813797	+	13	3	3	527	533	FBgn0086673	nonsynonymous
17	2L	17177434	17177435	+	7	69	69	746	747	FBgn0045487	frameshift
18	2L	18391190	18391193	+	5	38	40	1308	1311	FBgn0000636	nonsynonymous
19	2L	19544646	19544670	+	18	45	41	1199	1223	FBgn0003231	nonsynonymous
20	2L	19544646	19544670	+	18	45	41	1199	1223	FBgn0003231	nonsynonymous

Continued on next page

no.	chr	start	end	strand	w	conc. cov. 1	conc. cov. 2	CDS start	CDS end	Gene ID	Consequence
21	2L	3579416	3579419	-	5	9	9	761	764	FBgn0004892	nonsynonymous
22	2L	4733376	4733379	-	4	59	59	997	1000	FBgn0031626	nonsynonymous
23	2L	5321064	5321073	-	31	16	13	3676	3685	FBgn0031698	nonsynonymous
24	2L	5321064	5321073	-	31	16	13	3685	3694	FBgn0031698	nonsense
25	2L	6463609	6463648	-	4	2	8	196	235	FBgn0031816	nonsynonymous
26	2L	6530256	6530259	-	8	36	38	190	193	FBgn0000320	nonsynonymous
27	2L	6530256	6530259	-	8	36	38	172	175	FBgn0000320	nonsynonymous
28	2L	7132794	7132806	-	13	34	35	590	602	FBgn0085407	nonsynonymous
29	2L	7132794	7132806	-	13	34	35	590	602	FBgn0085407	nonsynonymous
30	2L	8447498	8447501	-	5	60	61	1689	1692	FBgn0027780	nonsynonymous
31	2L	8961234	8961240	-	4	78	69	1115	1121	FBgn0032080	nonsynonymous
32	2L	10000272	10000284	-	4	99	99	1184	1196	FBgn0051755	nonsynonymous
33	2LHet	168071	168083	-	8	101	99	592	604	FBgn0058042	nonsynonymous
34	2LHet	168071	168083	-	8	101	99	352	364	FBgn0058042	nonsynonymous
35	2R	4586016	4586018	+	13	57	57	1366	1368	FBgn0033321	frameshift
36	2R	4686607	4686640	+	5	6	1	38	71	FBgn0024189	nonsynonymous
37	2R	4686607	4686640	+	5	6	1	38	71	FBgn0024189	nonsynonymous
38	2R	8891058	8891061	+	10	18	19	3284	3287	FBgn0008654	nonsynonymous
39	2R	8891058	8891061	+	10	18	19	3284	3287	FBgn0008654	nonsynonymous
40	2R	9734854	9734863	+	12	20	22	497	506	FBgn0050484	nonsense
41	2R	9927935	9927941	+	5	11	12	2417	2423	FBgn0002643	nonsynonymous
42	2R	9927935	9927941	+	5	11	12	2417	2423	FBgn0002643	nonsynonymous
43	2R	10397874	10397877	+	10	30	32	4569	4572	FBgn0000142	nonsynonymous
44	2R	10397874	10397877	+	10	30	32	4569	4572	FBgn0000142	nonsynonymous
45	2R	12051906	12051909	+	5	1	2	175	178	FBgn0034083	nonsynonymous
46	2R	14017091	14017097	+	4	28	28	628	634	FBgn0040465	nonsynonymous
47	2R	19539490	19539493	+	13	1	1	1556	1559	FBgn0004795	nonsynonymous
48	2R	19539490	19539493	+	13	1	1	1571	1574	FBgn0004795	nonsynonymous
49	2R	20408468	20408497	+	4	47	44	1523	1552	FBgn0259187	frameshift
50	2R	7157457	7157466	-	10	13	11	325	334	FBgn0033603	nonsynonymous
51	2R	7157457	7157466	-	10	13	11	325	334	FBgn0033603	nonsynonymous
52	2R	8309740	8309746	-	6	79	80	188	194	FBgn0033733	nonsynonymous
53	2R	8309740	8309746	-	6	79	80	188	194	FBgn0033733	nonsynonymous
54	2R	8819857	8819860	-	7	12	12	1234	1237	FBgn0033783	nonsynonymous
55	2R	9950277	9950292	-	13	17	16	5155	5170	FBgn0040752	nonsynonymous
56	2R	10498192	10498201	-	21	63	63	6559	6568	FBgn0026427	nonsynonymous
57	2R	10498192	10498201	-	21	63	63	1687	1696	FBgn0026427	nonsynonymous
58	2R	11653223	11653226	-	4	4	4	5485	5488	FBgn0083919	nonsynonymous
59	2R	13053562	13053604	-	4	0	3	286	328	FBgn0034204	nonsynonymous
60	2R	17979419	17979449	-	5	28	33	2862	2892	FBgn0034693	nonsynonymous
61	2R	17979419	17979449	-	5	28	33	1056	1086	FBgn0034693	nonsynonymous
62	2R	18012854	18012866	-	8	46	48	1667	1679	FBgn0034697	nonsynonymous
63	2R	18012854	18012866	-	8	46	48	1787	1799	FBgn0034697	nonsynonymous
64	2R	18110325	18110326	-	9	78	78	656	657	FBgn0034715	frameshift
65	2R	19170797	19170800	-	9	11	11	634	637	FBgn0034840	nonsynonymous
66	2R	19625357	19625360	-	51	4	4	1000	1003	FBgn0000562	nonsynonymous
67	2R	19900210	19900211	-	4	43	43	55	56	FBgn0053519	frameshift
68	2R	20136710	20136713	-	4	30	28	2016	2019	FBgn0034994	nonsynonymous
69	2R	20313352	20313355	-	12	0	0	142	145	FBgn0259210	nonsynonymous
70	2R	20313352	20313355	-	12	0	0	142	145	FBgn0259210	nonsynonymous
71	2R	20313352	20313355	-	12	0	0	142	145	FBgn0259210	nonsynonymous
72	2R	20834457	20834460	-	5	73	75	1356	1359	FBgn0027599	nonsynonymous
73	2RHet	471528	471531	-	5	101	100	188	191	FBgn0043842	nonsynonymous
74	3L	1229685	1229688	+	7	27	27	83	86	FBgn0260963	nonsense
75	3L	1314504	1314513	+	5	22	25	276	285	FBgn0035207	nonsynonymous
76	3L	1314504	1314513	+	5	22	25	276	285	FBgn0035207	nonsynonymous
77	3L	5476159	5476168	+	8	38	38	3062	3071	FBgn0035607	nonsynonymous
78	3L	7238085	7238124	+	10	54	57	598	637	FBgn0016036	nonsynonymous
79	3L	9079370	9079400	+	15	66	74	3156	3186	FBgn0023479	nonsynonymous
80	3L	9079370	9079400	+	15	66	74	846	876	FBgn0023479	nonsynonymous
81	3L	9079390	9079420	+	12	71	78	3176	3206	FBgn0023479	nonsynonymous
82	3L	9079390	9079420	+	12	71	78	866	896	FBgn0023479	nonsynonymous
83	3L	9439363	9439375	+	5	27	27	596	608	FBgn0053926	nonsynonymous
84	3L	9586020	9586026	+	5	23	24	1238	1244	FBgn0261555	nonsynonymous
85	3L	9586020	9586026	+	5	23	24	1856	1862	FBgn0261555	nonsynonymous
86	3L	11210058	11210067	+	4	29	26	130	139	FBgn0261553	nonsynonymous
87	3L	11210058	11210067	+	4	29	26	130	139	FBgn0261553	nonsynonymous
88	3L	11210058	11210067	+	4	29	26	130	139	FBgn0261553	nonsynonymous
89	3L	11210058	11210067	+	4	29	26	130	139	FBgn0261553	nonsynonymous
90	3L	11210058	11210067	+	4	29	26	130	139	FBgn0261553	nonsynonymous
91	3L	12879687	12879720	+	5	1	1	1536	1569	FBgn0036321	nonsynonymous
92	3L	13488857	13488866	+	4	5	5	131	140	FBgn0036373	nonsynonymous
93	3L	13844314	13844315	+	13	38	38	3601	3602	FBgn0029167	frameshift
94	3L	14001788	14001797	+	4	52	50	839	848	FBgn0036398	nonsynonymous
95	3L	14227073	14227163	+	11	43	29	425	515	FBgn0036416	nonsynonymous
96	3L	14658844	14658845	+	23	2	2	794	795	FBgn0041604	frameshift
97	3L	16709753	16709756	+	4	72	76	1200	1203	FBgn0000414	nonsynonymous
98	3L	16709753	16709756	+	4	72	76	1200	1203	FBgn0000414	nonsynonymous
99	3L	22687535	22687553	+	4	9	9	334	352	FBgn0037179	nonsynonymous
100	3L	23709609	23709610	+	4	60	60	598	599	FBgn0039977	frameshift
101	3L	1435919	1435921	-	7	41	41	1544	1546	FBgn0043865	frameshift

Continued on next page

no.	chr	start	end	strand	w	conc. cov. 1	conc. cov. 2	CDS start	CDS end	Gene ID	Consequence
102	3L	1435919	1435921	-	7	41	41	1544	1546	FBgn0043865	frameshift
103	3L	6725421	6725427	-	8	52	53	5542	5548	FBgn0052394	nonsynonymous
104	3L	6744039	6744045	-	4	31	31	433	439	FBgn0035713	nonsynonymous
105	3L	6744039	6744045	-	4	31	31	433	439	FBgn0035713	nonsynonymous
106	3L	7230592	7230599	-	8	26	28	535	542	FBgn0035751	frameshift
107	3L	7824217	7824223	-	4	5	5	456	462	FBgn0016694	nonsynonymous
108	3L	7824217	7824223	-	4	5	5	456	462	FBgn0016694	nonsynonymous
109	3L	8985631	8985640	-	15	38	38	2871	2880	FBgn0016070	nonsynonymous
110	3L	8985631	8985640	-	15	38	38	2871	2880	FBgn0016070	nonsynonymous
111	3L	8985631	8985640	-	15	38	38	2871	2880	FBgn0016070	nonsynonymous
112	3L	8985631	8985640	-	15	38	38	2871	2880	FBgn0016070	nonsynonymous
113	3L	8985631	8985640	-	15	38	38	2871	2880	FBgn0016070	nonsynonymous
114	3L	9409723	9409726	-	4	2	2	448	451	FBgn0035988	nonsynonymous
115	3L	10216992	10217004	-	5	30	33	505	517	FBgn0052056	nonsynonymous
116	3L	12768985	12769003	-	13	111	117	1352	1370	FBgn0052106	nonsynonymous
117	3L	13511529	13511532	-	13	14	14	227	230	FBgn0036377	nonsynonymous
118	3L	13907404	13907407	-	10	23	23	222	225	FBgn0026376	nonsynonymous
119	3L	14018835	14018838	-	10	0	0	2671	2674	FBgn0052133	nonsynonymous
120	3L	16918205	16918223	-	4	14	12	862	880	FBgn0036677	nonsynonymous
121	3L	18663249	18663255	-	6	5	4	1765	1771	FBgn0042134	nonsynonymous
122	3R	3021473	3021482	+	23	28	29	1850	1859	FBgn0086372	nonsynonymous
123	3R	3021473	3021482	+	23	28	29	1850	1859	FBgn0086372	nonsynonymous
124	3R	9907419	9907425	+	9	33	33	1641	1647	FBgn0038197	nonsynonymous
125	3R	9907419	9907425	+	9	33	33	1641	1647	FBgn0038197	nonsynonymous
126	3R	9907419	9907425	+	9	33	33	2196	2202	FBgn0038197	nonsynonymous
127	3R	10342878	10342890	+	9	73	63	104	116	FBgn0085302	nonsynonymous
128	3R	16114233	16114236	+	8	9	11	290	293	FBgn0038780	nonsynonymous
129	3R	21168109	21168139	+	29	91	99	1733	1763	FBgn0027376	nonsynonymous
130	3R	23196662	23196665	+	10	35	35	4628	4631	FBgn0260487	nonsynonymous
131	3R	24526620	24526635	+	6	35	35	1243	1258	FBgn0027655	nonsynonymous
132	3R	24527322	24527331	+	23	8	8	1511	1520	FBgn0027655	nonsynonymous
133	3R	24735787	24735790	+	26	3	3	3886	3889	FBgn0259220	nonsynonymous
134	3R	24735787	24735790	+	26	3	3	247	250	FBgn0259220	nonsynonymous
135	3R	2217261	2217276	-	5	1	0	454	469	FBgn0037443	nonsynonymous
136	3R	2217261	2217276	-	5	1	0	454	469	FBgn0037443	nonsynonymous
137	3R	2217261	2217276	-	5	1	0	454	469	FBgn0037443	nonsynonymous
138	3R	2217261	2217276	-	5	1	0	40	55	FBgn0037443	nonsynonymous
139	3R	2217261	2217276	-	5	1	0	577	592	FBgn0037443	nonsynonymous
140	3R	4609852	4609858	-	11	83	86	832	838	FBgn0261015	nonsynonymous
141	3R	4609852	4609858	-	11	83	86	832	838	FBgn0261015	nonsynonymous
142	3R	4609852	4609858	-	11	83	86	1873	1879	FBgn0261015	nonsynonymous
143	3R	6598791	6598794	-	18	104	103	165	168	FBgn0020439	nonsynonymous
144	3R	6600757	6600763	-	6	23	23	100	106	FBgn0020439	nonsynonymous
145	3R	8227435	8227441	-	10	55	55	27	33	FBgn0038035	nonsynonymous
146	3R	10039846	10039861	-	4	14	14	139	154	FBgn0011582	nonsynonymous
147	3R	10039846	10039861	-	4	14	14	139	154	FBgn0011582	nonsynonymous
148	3R	10147775	10147778	-	6	58	58	2838	2841	FBgn0038223	nonsynonymous
149	3R	10580166	10580169	-	7	58	57	320	323	FBgn0038258	nonsynonymous
150	3R	11248525	11248531	-	6	49	51	1998	2004	FBgn0038339	nonsynonymous
151	3R	11248525	11248531	-	6	49	51	2250	2256	FBgn0038339	nonsynonymous
152	3R	13058895	13058912	-	4	63	52	1111	1128	FBgn0038494	frameshift
153	3R	13609108	13609120	-	4	19	19	529	541	FBgn0038544	nonsynonymous
154	3R	13609330	13609333	-	5	34	33	316	319	FBgn0038544	nonsynonymous
155	3R	13609345	13609348	-	8	32	34	301	304	FBgn0038544	nonsynonymous
156	3R	15196876	15196879	-	4	3	3	4514	4517	FBgn0086613	nonsynonymous
157	3R	25414838	25414841	-	21	78	79	644	647	FBgn0039671	nonsynonymous
158	X	5732240	5732252	+	30	1	5	1629	1641	FBgn0029814	nonsynonymous
159	X	6552220	6552232	+	4	1	0	1135	1147	FBgn0028360	nonsynonymous
160	X	8406521	8406536	+	5	0	0	650	665	FBgn0053181	nonsynonymous
161	X	8406521	8406536	+	5	0	0	650	665	FBgn0053181	nonsynonymous
162	X	8406521	8406536	+	5	0	0	140	155	FBgn0053181	nonsynonymous
163	X	8406521	8406536	+	5	0	0	650	665	FBgn0053181	nonsynonymous
164	X	8406521	8406536	+	5	0	0	599	614	FBgn0053181	nonsynonymous
165	X	9927751	9927754	+	4	3	6	3001	3004	FBgn0030171	nonsynonymous
166	X	13484375	13484378	+	42	0	0	1468	1471	FBgn0086674	nonsynonymous
167	X	18744396	18744408	+	12	0	3	1817	1829	FBgn0085451	nonsynonymous
168	X	20965495	20965498	+	15	1	1	484	487	FBgn0064123	nonsynonymous
169	X	1651253	1651260	-	4	20	25	64	71	FBgn0040898	frameshift
170	X	5555088	5555094	-	17	2	0	992	998	FBgn0259150	nonsynonymous
171	X	5555088	5555094	-	17	2	0	992	998	FBgn0259150	nonsynonymous
172	X	19438581	19438590	-	4	1	1	550	559	FBgn0052532	nonsynonymous
173	X	20024919	20024926	-	4	21	22	233	240	FBgn0260872	frameshift

End of the table
