# Supplementary material for: A Deterministic Analysis of Genome Integrity during Neoplastic Growth in Drosophila
Source: PLoS One. 2014 Feb 6;9(2):e87090. doi: 10.1371/journal.pone.0087090 (PMC3916295; doi:10.1371/journal.pone.0087090)
Supplement: Table S3 — PCR-based validation of small insertions (ID 1–10), deletions (ID 11–20; 25–26) and tandem duplications (ID 21–24; 27). Homozygous (Hom) and heterozygous (Het) events were selected. The ‘length’ corresponds to the size of the event. The ‘size on ref.’ shows the expected product size of PCR product as estimated from the reference genome. The ‘corrected size’ represents the expected product size in a sample containing the SV (for tandem duplications, this corresponds to the size expected from a single duplication event). small ins = small insertion; del = deletion; tan dup = tandem duplication; SV = structural variant; FWD = forward; REV = reverse. (PDF) [file pone.0087090.s014.pdf]

Supporting Information for: "A deterministic analysis of genome integrity during neoplastic growth in *Drosophila*" Table S3

Cem Sievers[1], Federico Comoglio[1], Makiko Seimiya[1], Gunter Merdes[1,*] and Renato Paro[1,2,*]

[1]Department of Biosystems Science and Engineering, Swiss Federal Institute of Technology Zurich, Mattenstrasse 26, 4058 Basel, Switzerland

[2]Faculty of Science, University of Basel, Klingelbergstrasse 50, 4056 Basel, Switzerland

July 12, 2013

ID	Coordinates	Genotype	SV type	Length	size on ref. (r)	corrected size (t)	FWD primer	REV primer
1	2L:18578669	Hom	small ins	120	196	316	CCTGGATACCTCTGGCAAAC	AGGGAGCCAGTTGAAATTGA
2	2L:21017450	Hom	small ins	133	183	316	TCGGATTAATTCGCTTTATGA	CAGATGCTGATAAGGGAAAGATG
3	3R:1975276	Hom	small ins	100	127	227	TTTTCTCAGTGCATACTCAGCAG	CTGCCCAACTAAAGCAAAGG
4	2L:2646518	Hom	small ins	102	149	251	GGATGCTGTCATTTGCTACG	CACAAACTGCCAGCTACCAA
5	2R:4530459	Het	small ins	114	140	254	TTCAATCTCCTGTGTGCTGAA	CCCAGAAAAATAGGCACTTTC
6	3R:21701316	Het	small ins	114	164	278	AATTATGATTCGGGCACACC	CCATTGAACAACGCCATTTA
7	2L:3289401	Hom	small ins	25	146	171	TTTTCAAAGCAAAAGCAGTCC	ATCGGCTGTAGGTCCTTGAA
8	2R:3872130	Hom	small ins	22	124	146	GATTGGGAGGAAATGCTGAA	TGATTATGATAGGCTAAGCAAGC
9	3L:17484346	Hom	small ins	28	188	216	AGTCGCCACTATCAGCCAGA	TGCACAAATATGCTTCCGTTA
10	3R:1771634	Het	small ins	31	181	212	ACTTTTTCCACTGGGTCCAT	GAAGGCATAAAATAGAAAGGCAAA
11	3R:8492543	Hom	del	74	251	177	CGATCAAAGGCCATCAAAAT	TGCTTTGTAGATAGTGTCATTAAAAGG
12	3L:10182662	Hom	del	132	298	166	ATGAAAACTGCAAGCGACCT	TGCTTAAGCCCTTTTTCGTT
13	3L:22637989	Het	del	175	317	142	GCCAAATATGGAGTGCGAGT	TCGTTGTTGAATTTATGGGTGA
14	3R:26987829	Het	del	245	368	123	AATTTGGCTGCGAATGAAAG	TGGCAAATGTTTGAGTTTGG
15	3R:22289554	Het	del	120	316	196	GCCTTCCACAGTTCATTGCT	GGCTGTTTCGTAATGATCCA
16	2L:12736726	Hom	del	23	200	177	CTGATCGGTACCCGGAAAAT	TTGGTTAGTCACAGCGAACG
17	2L:5757822	Hom	del	22	200	178	GCGTAATTAGTTTCAACCAGTGC	TTTGGCATTTAAAACAGAACG
18	3R:3765275	Het	del	22	146	124	TTCTTGTTCGCAAATAAACCA	TCCTGAATTTGTACATATATTTAATCG
19	2R:8489223	Het	del	25	202	177	TGCTGGGTATGAAAACAAAGG	AAAATGCATATTTCGGTGCT
20	2R:10867320	Het	del	23	206	183	AGCTGAATTTAGAAGTTGTGGTG	ACAAAAGTATACATGGTTTGTCCA
21	3L:15041213	n.d.	tan dup	418	495	913	TTTCCCATAAAGTATGGTCAATAAAA	TGACATAGGTCCAATGGTTTCA
22	3R:20192157	n.d.	tan dup	565	648	1213	CTGGCACACAGTTGCAGTTT	GATTGGGGTCTCCAGATGAA
23	2R:5543741	n.d.	tan dup	889	985	1874	GCCAGGATGGGAATTTTCTT	TGGCACTAAAATGCAAATGG
24	2L:3026214	n.d.	tan dup	710	773	1483	TAAGGAGGATCGGGAGAGGT	CAAGTGCTGCCTGAGAAGC
25	3L:15825407	Het	del	28	205	177	CGAACCCGATTGTTTTTGTT	GCCTGAAGTTTCTCCACGTC
26	3L:15828140	Het	del	102	215	113	CTTTCTCCCACACAGCTGAA	TAGGTAAGCAGCGGTGGATT
27	3L:15826899	n.d.	tan dup	293	442	735	TGGGATGTTGCCAAATGTTA	CTAAGGGCCCAGCTGAATTT
