# Supplementary material for: A Deterministic Analysis of Genome Integrity during Neoplastic Growth in Drosophila
Source: PLoS One. 2014 Feb 6;9(2):e87090. doi: 10.1371/journal.pone.0087090 (PMC3916295; doi:10.1371/journal.pone.0087090)
Supplement: Table S4 — Simulation results. 1000 structural variations of the specified type were generated. refers to the total number of recalled events, whereas refers to the tolerance used to evaluate SV calls with respect to the genomic coordinates of the generated SV. For translocations marked with , the number of insertional duplications are indicated in parenthesis as they correspond to events matching a partial signature. BD = BreakDancer. (PDF) [file pone.0087090.s015.pdf]

Supplementary material for: "A deterministic analysis of genome integrity during neoplastic growth in *Drosophila*"
Table S4

Cem Sievers[1], Federico Comoglio[1], Makiko Seimiya[1], Gunter Merdes[1,*] and Renato Paro[1,2,*]

[1]Department of Biosystems Science and Engineering, Swiss Federal Institute of Technology Zurich, Mattenstrasse 26, 4058 Basel, Switzerland

[2]Faculty of Science, University of Basel, Klingelbergstrasse 50, 4056 Basel, Switzerland

December 14, 2013

	DSVD			Delly-PE			BD	Pindel	CLEVER
	$c = 1$x	$c = 5$x	$c = 20$x	$c = 1$x	$c = 5$x	$c = 20$x	$c = 20$x	$c = 20$x	$c = 20$x
**Single and double breakpoint events**									
Small insertions									
n	228	704	971	-	-	-	288	-	-
±0	170	491	674	-	-	-	-	-	-
±1	223	673	915	-	-	-	-	-	-
±2	226	692	943	-	-	-	-	-	-
±5	226	694	948	-	-	-	-	-	-
Small deletions									
n	215	745	997	-	-	-	368	154	-
±0	158	514	675	-	-	-	-	-	-
±1	206	714	925	-	-	-	-	-	-
±2	211	733	955	-	-	-	-	-	-
±5	211	733	958	-	-	-	-	-	-
Large deletions									
n	250	718	980	185	858	966	950	963	960
±0	180	539	696	182	778	668	-	-	-
±1	239	692	920	184	791	715	-	-	-
±2	243	707	939	184	805	784	-	-	-
±5	244	710	944	185	847	940	-	-	-
Inversions									
n	394	889	953	27	731	957	954	977	-
±0	288	664	686	27	731	957	-	-	-
±1	387	874	936	27	731	957	-	-	-
±2	394	887	949	27	731	957	-	-	-
±5	394	889	953	27	731	957	-	-	-
Tandem Duplications									
n	229	708	951	196	874	975	-	965	-
±0	171	514	683	181	784	656	-	-	-

Continued on next page

	DSVD			Delly-PE			BD	Pindel	CLEVER
	$c = 1x$	$c = 5x$	$c = 20x$	$c = 1x$	$c = 5x$	$c = 20x$	$c = 20x$	$c = 20x$	$c = 20x$
± 1	225	695	933	185	802	716	-	-	-
± 2	228	708	946	187	820	779	-	-	-
± 5	229	708	951	195	864	941	-	-	-
**Insertional duplications**									
intrachromosomal downstream no inversion									
n	57	500	911	-	-	-	-	-	-
± 0	52	458	830	-	-	-	-	-	-
± 1	56	498	900	-	-	-	-	-	-
± 2	57	500	900	-	-	-	-	-	-
± 5	57	500	910	-	-	-	-	-	-
intrachromosomal downstream inversion									
n	53	519	915	-	-	-	-	-	-
± 0	49	471	829	-	-	-	-	-	-
± 1	52	514	896	-	-	-	-	-	-
± 2	52	515	898	-	-	-	-	-	-
± 5	53	519	915	-	-	-	-	-	-
intrachromosomal upstream no inversion									
n	53	499	914	-	-	-	-	-	-
± 0	48	455	837	-	-	-	-	-	-
± 1	53	497	906	-	-	-	-	-	-
± 2	53	497	910	-	-	-	-	-	-
± 5	53	498	913	-	-	-	-	-	-
intrachromosomal upstream inversion									
n	50	490	907	-	-	-	-	-	-
± 0	47	460	838	-	-	-	-	-	-
± 1	50	489	900	-	-	-	-	-	-
± 2	50	490	902	-	-	-	-	-	-
± 5	50	490	907	-	-	-	-	-	-
interchromosomal no inversion									
n	45	505	891	-	-	-	-	-	-
± 0	40	462	803	-	-	-	-	-	-
± 1	44	504	888	-	-	-	-	-	-
± 2	44	505	889	-	-	-	-	-	-
± 5	45	505	891	-	-	-	-	-	-
interchromosomal inversion									
n	34	489	893	-	-	-	-	-	-
± 0	29	453	820	-	-	-	-	-	-
± 1	34	487	887	-	-	-	-	-	-
± 2	34	487	888	-	-	-	-	-	-
± 5	34	488	892	-	-	-	-	-	-
**Translocations**									
intrachromosomal downstream									

Continued on next page

	DSVD			Delly-PE			BD	Pindel	CLEVER
	$c = 1$x	$c = 5$x	$c = 20$x	$c = 1$x	$c = 5$x	$c = 20$x	$c = 20$x	$c = 20$x	$c = 20$x
no inversion									
n	13	374	901	-	-	-	-	-	-
± 0	13	339	811	-	-	-	-	-	-
± 1	13	374	900	-	-	-	-	-	-
± 2	13	374	901	-	-	-	-	-	-
± 5	13	374	901	-	-	-	-	-	-
intrachromosomal downstream inversion									
n	11	380	855	-	-	-	-	-	-
± 0	10	345	794	-	-	-	-	-	-
± 1	11	379	873	-	-	-	-	-	-
± 2	11	380	878	-	-	-	-	-	-
± 5	11	380	884	-	-	-	-	-	-
intrachromosomal upstrem no inversion									
n	11	381	914	-	-	-	-	-	-
± 0	10	357	836	-	-	-	-	-	-
± 1	11	381	914	-	-	-	-	-	-
± 2	11	381	914	-	-	-	-	-	-
± 5	11	381	914	-	-	-	-	-	-
intrachromosomal upstream inversion									
n	13	385	909	-	-	-	-	-	-
± 0	11	362	824	-	-	-	-	-	-
± 1	13	385	904	-	-	-	-	-	-
± 2	13	385	909	-	-	-	-	-	-
± 5	13	385	909	-	-	-	-	-	-
interchromosomal no inversion†									
n	20(46)	387(121)	901(10)	28	750	979	528	-	-
± 0	20	361	804	28	750	979	-	-	-
± 1	20	387	901	28	750	979	-	-	-
± 2	20	387	901	28	750	979	-	-	-
± 5	20	387	901	28	750	979	-	-	-
interchromosomal inversion†									
n	10(41)	392(134)	895(10)	22	774	974	144	-	-
± 0	10	360	779	22	774	974	-	-	-
± 1	10	391	885	22	774	974	-	-	-
± 2	10	391	888	22	774	974	-	-	-
± 5	10	392	895	22	774	974	-	-	-
