# Supplementary material for: A Deterministic Analysis of Genome Integrity during Neoplastic Growth in Drosophila
Source: PLoS One. 2014 Feb 6;9(2):e87090. doi: 10.1371/journal.pone.0087090 (PMC3916295; doi:10.1371/journal.pone.0087090)
Supplement: Table S5 — DNA sequences of selected SVs obtained through Sanger sequencing of the PCR products indicated Figure S8. SV sequences (reported in the second column) are indicated in blue. Deletions are additionally crossed by a line. Flanking sequences are shown in black. Bases in red correspond to the 5 positions adjacent to the SV. Sequencing results (third column) show the Sanger sequencing results. Red bases across columns indicate corresponding positions. (PDF) [file pone.0087090.s016.pdf]

Supporting Information for:
"A deterministic analysis of genome integrity during neoplastic growth in *Drosophila*"
Table S5

Cem Sievers[1], Federico Comoglio[1], Makiko Seimiya[1], Gunter Merdes[1,*] and Renato Paro[1,2,*]

[1]Department of Biosystems Science and Engineering, Swiss Federal Institute of Technology Zurich, Mattenstrasse 26, 4058 Basel, Switzerland

[2]Faculty of Science, University of Basel, Klingelbergstrasse 50, 4056 Basel, Switzerland

December 14, 2013

ID	SV sequence	Sequencing result
1	CCTGGATACCTCTGGCAAACAGCTTGCTATCGCTCA<br>CCCACACACATGACCTTCGGTGGTTGCCACCACCCC<br>CATGGGTTTTTCTCATATGAGCAACTCACTACCCGG<br>CAAGAAGGCCATAAAAGCAAATGAAAGCGGATGGAT<br>ACCGAGTGACCAGCGAATCCCAGCTCGCTTGAACTT<br>AATGCGCTATTAGCCAACTTTATTCGGACCATGTTG<br>TTAGCCGGCTAGTGGCGAGCAACCCAATGCGGTTAT<br>TTGTTATGCTACAAATTAGCCTGGTCAATTAGCGTT<br>GGCCTGATTCAATTTCAACTGGCTCCCTCGGGG	ATGGGTTTTTCTCATATGAGCAACTCACTACCCGGC<br>AAGAAGGCCATAAAAGCAAATGAAAGCGGATGGATA<br>CCGAGTGACCAGCGAATCCCAGCTCGCTTGAACTTA<br>ATGCGCTATTAGCCAACTTTATTCGGACCATGTTGT<br>TAGCCGGCTAGTGGCGAGCAACCCAATGCGGTTATT<br>TGTTATGCTACAAATTAGCCTGGTCAATTAGCGTTG<br>GCCTGATTCAATTTCAACTGG
2	CAAATAAATCGGATTAATTCGCTTTATGAAAGCATT<br>CAATTGTGAAATGAAATTTGATTCACAAAACACCGA<br>AATTTGAAGCATAAACTAATCATGCCCAACCGCACA<br>AATTTGTCTATTACATCTTATATACGTATTTCCTTCA<br>AATGTTCCAACTTTTTTGAACTTAATATTCGCGTAT<br>TAAGCACTTAAATCCCAAGAAAATATAATCCCTTAT<br>GTTTGGGTTTCTTGCAAATACCACAAAAACCGAGAG<br>GTAAAATTAAACAATTTAAGGCAAATTTAGTGACTG<br>TGTCTTCGTTTTCATCTTTCCCTTATCAGCATCTGAT<br>TTGTCAAC	TCATGCCCAACCGCACAAATTTGTCTATTACATCTTA<br>TATACGTATTTCCTTCAAATGTTCCAACTTTTTTGA<br>ACTTAATATTCGCGTATTAAGCACTTAAATCCCAAG<br>AAAATATAATCCCTTATGTTTGGGTTTCTTGCAAAT-<br>ACCACAAAAACCGAGAGGTAAAATTAAACAATTTAA<br>GGCAAATTTAGTGACTGTGTCTTCGTTTTCATCTTT<br>CCCTTANCAGCAT
3	TTCTGGGAACTCATTTAAAAAGATTTTTTTTTATCT<br>TTTCTACCTAATGACCATTTTTTCTCAGTGCATACTC<br>AGCAGACTTGATGCATAAGCCACTGGCTGAGGTCCG<br>GACTGAAAAGCAATCGTTGCTACAGCGCTCGTGTGC<br>ATACATACATCGTACATGGGGCCTACAGGGAGCTAG<br>ACCCTCTATAATATTTAACTTTTTCCTGAAATATACA<br>CTCTTTGTCTTGGCCAGCTCTGCGGTGGCATAGATT<br>TGGCTGCTCCTTTGCTTTAGTTGGGCAGTAAGTCAC<br>TTTCCCAGAAG	CGTTGCTACAGCGCTTCGTGTGCATACATACATCGT<br>ACATGGGGCCTACAGGGNGCTAGACCCTCTATAATA<br>TTTAACTTTTTCCTGAAATATACACTCTTTGTCTTGG<br>CCAGCTCTGCGGTGGCATAGATTTGGCTGCTCCTTT<br>GCT
4	GGTGTCAGTTTAATTGCAAACGTAAATTGGATGCTG<br>TCATTTGCTACGTTTTCATTTGTTCGAAAGTTGCTC<br>GGCCCTCCCGTTTCCAGATGGAATCTGTGCAGCATG<br>GCACCCCTGGCTGTTGCCATTGACTTTTGCTGCCAT<br>CCGTTCTGACGGACACTTCTGCCAGCTGCTCCAGAA<br>TCAGAATCTCAGCTCACAGCTCGAATCTACCCGGAA<br>TGCAAACGTTGCACAAATGCCAGTTTTGTTAATTGG<br>ACTCATTTTGGTAGCTGGCAGTTTGTGTATTTCAAA<br>ATTGGCATTTGTGAC	TCTGTGCAGCATGGCACCCCTGGCTGTTGCCATTGA<br>CTTTTGCTGCCATCCGTTCTGACGGACACTTCTGCC<br>AGCTGCTCCAGAATCAGAATCTCAGCTCACAGCTCG<br>AATCTACCCGGAATGCAAACGTTGCACAAATGCCAG<br>TTTTGTTAATTGGACTCATTTTGGTAGCTGGCAGTT<br>TGTGAANTGAAGNANANANNTCNCGNNACGTTTGCA<br>TTCGGGTGATTGNGCTGTGAGCTGAGATTCTGATTC<br>TGGAGCA
7	GAATAAATTCAGACTTTCCTAGCTGGATAAGCTCTA<br>CAATTTTCAAAGCAAAAGCAGTCCTTCAATAAAATA<br>AATTAACCCTGTTTATACAAAATGTCCACTTGTTGG<br>GCTTTTTGTATATGTACGAACGAAAAAATTCTCGTT<br>TAATTGCTCAAAGGAAAATTAATTAGAGTGGCAATT<br>TTTTACAAGTTTCAAGGACCTACAGCCGATATAAAT<br>CTCAATAGGA	AAATGTCCACTTGTTGGGCTTTTTGTATATGTACG<br>AACGAAAAAATTCTCGTTTAATTGCTCAAAGGAAAA<br>TTAATTAGAGTGGCAATTTTTTACAAGTTTCAAGGA<br>CCTACAGCCGAT
8	CAAAAAGTCCTAATCTGCAAGCATTATGAAGATAAG<br>ATTGGGAGGAAATGCTGAACTGAATCAATTAAACTA<br>GATTAAGCGCTATTTGTTATTGGATTTCTGAGTTAG<br>ATCCCTAAAGCTGTGAATTTAAATCAAAGAAAACAT<br>GATGATATACAATAGCTTGCTTAGCCTATCATAATC<br>ATATGAATAACAAACTAGATTATTAAAAGTCCTCAT<br>ATATATC	TTGTTATTGGATTTCTGAGTTAGATCCCTAAAGCTG<br>TGAATTTAAATCAAAGAAAACATGATGATATACAAT<br>AGCTTGCTTAGCCTATCATAATCAAAAA
9	AACTAACTTTAGTCGCCACTATCAGCCAGATGTTGG<br>CCTTACATTATGAATAACTCAAACGGATTTAGCAGT<br>AGTCCCAAAAGATACAGAAAATGCATGCACAACTAA<br>CTTAAACTTGTAAAGATACAAATGTATCTTGCTAATT<br>TTCTCACTTTAAGAACTGAAATAAGGGATAGTACTC<br>ATCGTATGATTACAAGATTATTTCTAACGGAAGCAT<br>ATTTGTGCAAAT	ACAGAAAATGCATGCACAACTAACTTAAACTTGTAA<br>AGATACAAATGTATCTTGCTAATTTTCTCACTTTAAG<br>AACTGAAATAAGGGATAGTACTCATCGTATGATTAC<br>AAGATTATTTCTAACGGAAGCATATTTGTGCANANN<br>NTAANAATATCATATTCNCAGNCGCNTTTTGGNCTT<br>NTATTATGAATCCTCTNNGATTTAGACTANTCCAAA<br>ANATANGAAAATGCATGCAAACTAACTTAAATNGTA<br>AAGATAAAATGTATCTTGCTAATTTTCTCCTTTAAG<br>AACTGAAATAAGGGATAGTACTCATNTATGATTACA<br>ANATTATTTCTAACGGANGCATATT
11	ACTCGAGAAACAAACAACTCAGCCGATCAAAGGCCA<br>TCAAAATTAAAATCTCGTATGGAAAATGGAAAGTTT<br>GATGCAAACGTAGAGATAGATTTTTACGG~~CGGCTGG~~<br>~~CTCAAAAGTGTTTTATTAGTGAAATGGAAACGGACT~~<br>~~TTTAATTGAAATCATCTTGAATGAAGAGCGG~~TGGTA<br>ATGTGTTGATTTCATTTCGGTGGCCCAGCCAAAGAT<br>CATAAAAGCCATCATGCATGCATGCATCCCCCCTTT<br>TAATGACACTATCTACAAAGCAT	ATGCAACGTAGAGATAGATTTTTACGGTGGTAATGT<br>GTTGATTTCATTTCGGTGGCCCAGCCAAAGATCATA<br>AAAGCCATCATGCATGCATGCATCCCCCCTTTTAAT<br>GACACTATCTACAAAGCAAAAGT
17	CAAAGCGTAATTAGTTTCAACCAGTGCAATTGCAGA<br>TGACATCTTACATGGTGAATAAAAATTTAGCTATTT<br>TTTTATTCCGTTTTACCATATACTATATT~~TTAAATGT~~<br>~~TAAACATAAATATT~~GGTATAGATTCGATCTGCATTT<br>TTAAAGCAATTAAGTAAATAGGAAGGATAACATGCC<br>TACGTTCTGTTTTAAATGCCAAATGTAATAAATATT<br>TCAAAA	TATACTATATTGGTATAGATTCGATCTGCATTTTTAA<br>AGCAATTAAGTAAATAGGAAGGATAACATGCCTACG<br>TTCTGTTTTAAATGCCAAAAN
