# Supplementary material for: A Deterministic Analysis of Genome Integrity during Neoplastic Growth in Drosophila
Source: PLoS One. 2014 Feb 6;9(2):e87090. doi: 10.1371/journal.pone.0087090 (PMC3916295; doi:10.1371/journal.pone.0087090)
Supplement: Text S1 — Simulation of SVs for comparison of DSVD with other SV detection algorithms. (PDF) [file pone.0087090.s017.pdf]

Supplementary material for: “A deterministic analysis of genome integrity during neoplastic growth in *Drosophila*” Text S1

Cem Sievers[1], Federico Comoglio[1], Makiko Seimiya[1], Gunter Merdes[1,*] and Renato Paro[1,2,*]

[1]Department of Biosystems Science and Engineering, Swiss Federal Institute of Technology Zurich, Mattenstrasse 26, 4058 Basel, Switzerland

[2]Faculty of Science, University of Basel, Klingelbergstrasse 50, 4056 Basel, Switzerland

December 2, 2013

Detection of simulated SVs

The performance of DSVD was evaluated using simulated data encompassing the following 16 different types of structural variations (SVs):

1. Small insertions (of length $l \sim \mathcal{N}(15, 3)$, where $\mathcal{N}(\mu, \sigma)$ is a Normal distribution with mean μ and standard deviation σ.)
2. Small deletions ($l \sim \mathcal{N}(15, 3)$)
3. Deletions ($l \sim \mathcal{N}(1000, 100)$)
4. Inversions ($l \sim \mathcal{N}(1000, 100)$)
5. Tandem duplications ($l \sim \mathcal{N}(1000, 100)$)
6. Insertional duplications ($l \sim \mathcal{N}(1000, 100)$) according to the following 6 subtypes:
   (a) intra-chromosomal, downstream insertion without inversion
   (b) intra-chromosomal, downstream insertion with inversion
   (c) intra-chromosomal, upstream insertion without inversion
   (d) intra-chromosomal, upstream insertion with inversion
   (e) inter-chromosomal, insertion without inversion
   (f) inter-chromosomal, insertion with inversion
7. Translocations ($l \sim \mathcal{N}(1000, 100)$) of the same subtypes listed above for insertional duplications.

Where applicable, we compared our algorithm to the recently published SV detection algorithms DELLY (version 0.0.9) [6], BreakDancer (version 1.0) [7], Pindel (version 0.2.4) [8] and CLEVER (version 2.0) [9].

Simulation of SVs and SV detection using DSVD Chromosome 3R of the *Drosophila* reference genome (dm3) was used as context for all simulations. In addition, chromosome 2R was used as donor sequence for inter-chromosomal events. For each SV type, 1000 non-overlapping events were generated within the context sequence, yielding an aberrant chromosome 3R sequence containing all SVs. Next, wgsim 0.3.1 [10] was used to generate overlapping 150 nt paired-end reads with the following parameters: `-e 0.01 -R 0.001 -d 260 -s 15 -1 150 -2 150` (260 nt outer distance, 15 nt standard deviation, 1% base error

rate and indel fraction of 0.001). Reads were generated with a coverage of 1x, 5x and 20x for each simulation. DSVD was then used to call SVs and the algorithm calls were compared with the generated SVs. A call was considered positive if breakpoints coordinates were matched exactly at single base resolution. In addition, a second evaluation allowing a tolerance of ±1, ±2 and ±5 bp to the breakpoint coordinates was considered in order to account for equivalent alignments (see Methods in the main text).

Comparison with DELLY Since overlapping paired-end reads do not allow a fair comparison with DELLY, reads were trimmed to a length of 30nt (the seed length used by DSVD) using FASTX trimmer 0.0.13 with parameter `-t 120` and subsequently aligned to the dm3 genome using BWA 0.6.2 [11] with default parameters. The alignment output was converted to BAM format using SAMtools 0.1.18 and used as an input for DELLY (paired-end mode). A call was considered positive if the breakpoint-containing genomic intervals reported by the algorithm agreed with the generated SVs. This depends on the SV type and might imply fully contained, fully containing or overlapping intervals with respect to the generated events. For example, a deletion reported by DELLY was regarded as positive call if the simulated SV was entirely contained within the reported genomic interval. Note that, in contrast to DSVD, we do not require DELLY to identify SVs at single base resolution.

Comparison with BreakDancer We furthermore compared DSVD with BreakDancer, a paired-end read detection algorithm. The same input reads as used for the DELLY comparison were used. A coverage of 20x was considered for each SV type. Reads were aligned using MAQ with default parameters [12]. BreakDancer was run with default parameters and a call was considered positive if overlapping with the generated SV by at least 1 base.

Comparison with Pindel To compare DSVD with a split-read based SV detection algorithm, reads were trimmed to a length of 100nt using FASTX trimmer 0.0.13 with parameter `-t 50`, aligned with BWA, converted to BAM format, sorted and indexed. Pindel was run using default parameters and a call was considered positive if overlapping with the generated SV by at least 1 base. A coverage of 20x was considered for each SV type.

Comparison with CLEVER Comparison with CLEVER was performed as described above for Pindel, using reads trimmed to 30nt.

Simulation Results In the following, we report the number of called SVs for each SV type and subtype for DSVD and where applicable, for the other tested SV detection algorithms.

References

[1] Saj A, et al. (2010) A Combined Ex Vivo and In Vivo RNAi Screen for Notch Regulators in *Drosophila* Reveals an Extensive Notch Interaction Network. *Dev Cell* 18: 862-876.

[2] Beuchle,D., Struhl,G. and Mueller,J. (2001) Polycomb group proteins and heritable silencing of *Drosophila* Hox genes. *Development* **128**: 993-1004

[3] Xi,R., Hadjipanayis,A.G., Luquette,L.J., Kim,T., Lee,E., Zhang,J., Johnson,M.D, Muzny,D.M., Wheeler,D.A., et al. (2011) Copy number variation detection in whole-genome sequencing data using the Bayesian information criterion. *Proc Natl Acad Sci* **108**: E1128-1136. doi: 10.1073/pnas.1110574108.

[4] Li,H. (2011) A statistical framework for SNP calling, mutation discovery, association mapping and population genetical parameter estimation from sequencing data. *Bioinformatics* **27**: 2987-2993.

[5] Griffiths AJF, Wessler SR, Lewontin RC, Carroll SB (2008) *Introduction to genetic analysis*, 9th edition. W.H. Freeman and Company, New York, New York

[6] Rausch,T., Zichner,T., Schlattl,A., Stuetz,A.M., Benes,V. and Korbel,J.O. (2012) DELLY: structural variant discovery by integrated paired-end and split-read analysis. *Bioinformatics* **28**: i333-i339.

[7] Chen K, Wallis JW, McLellan MD, Larson DE, Kalicki JM, et al. (2009) BreakDancer: an algorithm for high-resolution mapping of genomic structural variation. *Nat Methods* 6: 677-781.

[8] Ye K, Schulz MH, Long Q, Apweiler R, Ning Z (2009) Pindel: a pattern growth approach to detect break points of large deletions and medium sized insertions from paired-end short reads. *Bioinformatics* 25: 2865-2871.

[9] Marschall T, Costa IG, Canzar S, Bauer M, Klau GW et al. (2012) CLEVER: clique-enumerating variant finder. *Bioinformatics* 28: 2875-2882.

[10] Li,H., Handsaker,B., Wysoker,A., Fennell,T., Ruan,J., Homer,N., Marth,G., Abecasis,G., Durbin,R. and the 1000 Genome Project Data Processing Subgroup (2009) The Sequence Alignment/Map format and SAMtools. *Bioinformatics* **25**: 2078-2079

[11] Li,H. and Durbin,R. 2010. Fast and accurate long-read alignment with Burrows-Wheeler Transform. *Bioinformatics* **26**: 589-595

[12] Li H, Ruan J, Durbin R (2008) Mapping short DNA sequencing reads and calling variants using mapping quality scores. *Genome Res* 18: 1851-1858.
